# Supplementary figures and images for: Combining transgenesis with paratransgenesis to fight malaria
Source: eLife. 2022 Oct 25;11:e77584. doi: 10.7554/eLife.77584 (PMC9596157; doi:10.7554/eLife.77584)

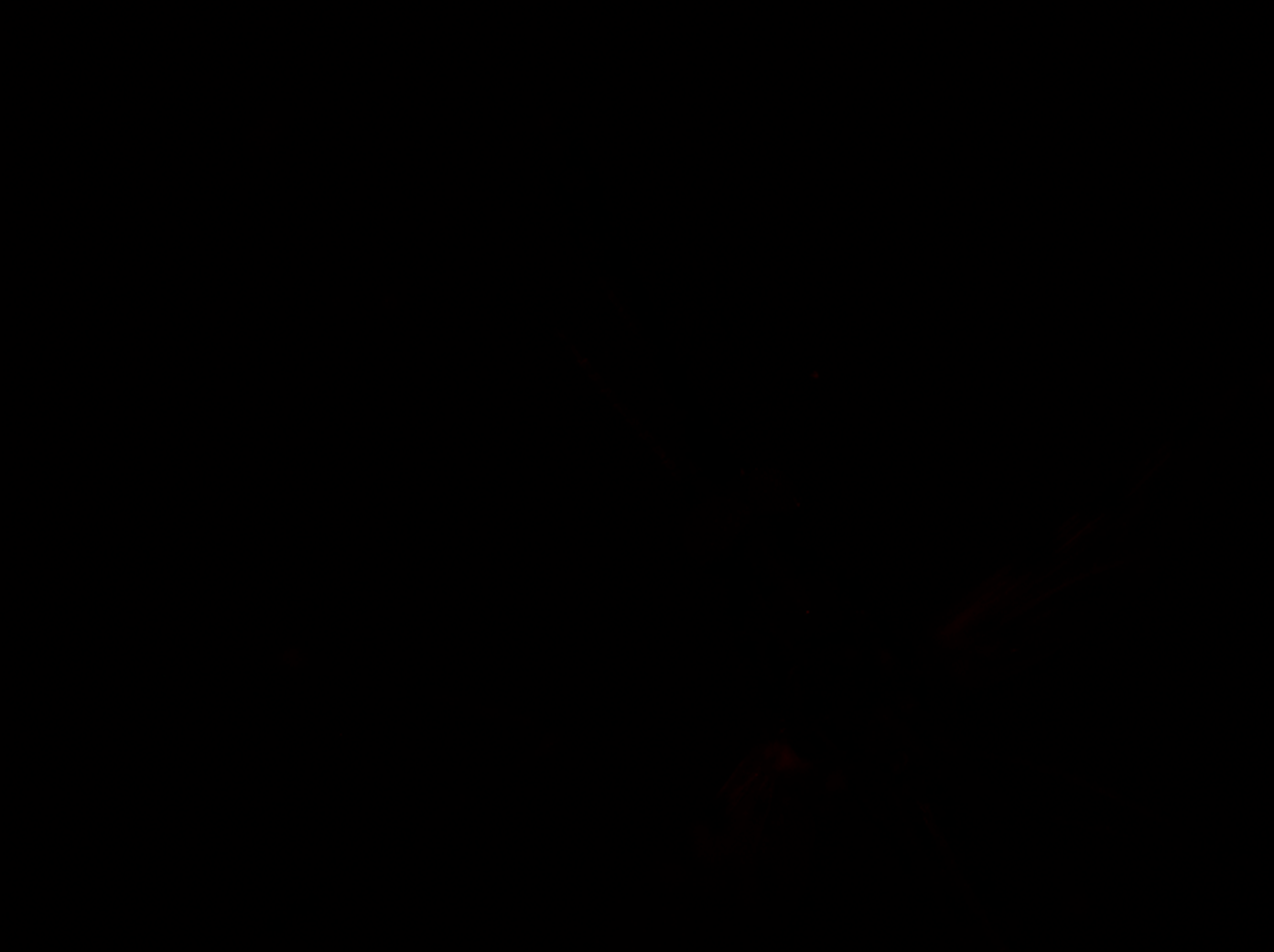

Supplement: Figure 1—source data 1. — Pictures of ‘Figure 1B-WT-blue field.tif,’ ‘Figure 1B-WT-red field.tif,’ and ‘Figure 1B-WT-yellow field.tif’ are original images of WT mosquito eye through blue, red, and yellow fluorescent filter, respectively; pictures of ‘Figure 1B-Mg-blue field.jpg,’ ‘Figure 1B-Mg-red field.jpg,’ and ‘Figure 1B-Mg-yellow field.jpg’ are original images of Mg mosquito line eye through blue, red, and yellow fluorescent filter, respectively; pictures of ‘Figure 1B-Sg-blue field.jpg,’ ‘Figure 1B-Sg-red field.jpg,’ and ‘-Figure 1B-Sg-yellow field.jpg’ are original images of Sg mosquito line eye through blue, red, and yellow fluorescent filter, respectively; pictures of ‘Figure 1B-E-blue field.jpg,’ ‘Figure 1B-E-red field.jpg,’ and ‘Figure 1B-E-yellow field.jpg’ are original images of E mosquito line eye through blue, red, and yellow fluorescent filter, respectively; pictures of Figure 1B-Mg-E-blue field.jpg, Figure 1B-Mg-E-red field.jpg, and Figure 1B-Mg-E-yellow field.jpg are original images of Mg/E mosquito line eye through blue, red, and yellow fluorescent filter, respectively; pictures of ‘Figure 1B-Sg-E-blue field.jpg,’ ‘Figure 1B-Sg-E-red field.jpg,’ and ‘Figure 1B-Sg-E-yellow field.jpg’ are original images of Sg/E mosquito line eye through blue, red, and yellow fluorescent filter, respectively; pictures of ‘Figure 1B-Mg+Sg-E-blue field.jpg,’ ‘Figure 1B-Mg+Sg-E-red field,’ and ‘Figure 1B-Mg+Sg-E-yellow field’ are original images of Mg/Sg/E mosquito line eye through blue, red, and yellow fluorescent filter, respectively. ‘Figure 1C and D-source data-RT-PCR data.xlsx’ is the original data for Figure 1C and D; ‘Figure 1C and D-gene expression.pzf’ shows Figure 1C and D were generated with GraphPad Prism. Pictures of ‘Figure 1E-western blot-MP2 in midgut.tif,’ ‘Figure 1E-western blot-Scorpine in midgut.tif,’ and ‘Figure 1E-western blot-α-tubulin in midgut.tif’ are original image of Western blots detected with mouse anti-MP2, mouse anti-scorpine, and rabbit anti-α-tubulin antibody. P [file elife-77584-fig1-data1.zip › Fig 1-source data/Fig1B-WT-red field.tif]

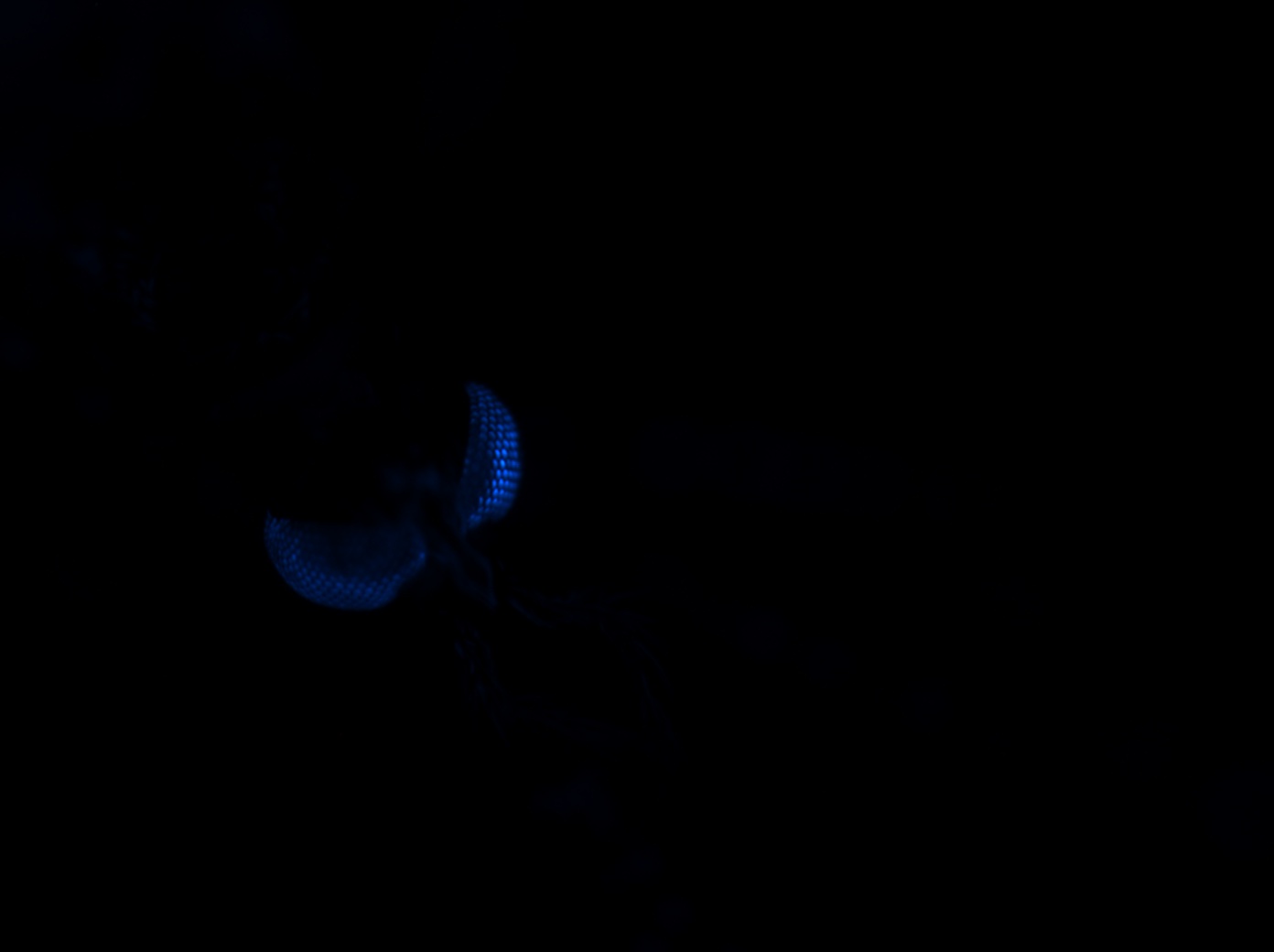

Supplement: Figure 1—source data 1. — Pictures of ‘Figure 1B-WT-blue field.tif,’ ‘Figure 1B-WT-red field.tif,’ and ‘Figure 1B-WT-yellow field.tif’ are original images of WT mosquito eye through blue, red, and yellow fluorescent filter, respectively; pictures of ‘Figure 1B-Mg-blue field.jpg,’ ‘Figure 1B-Mg-red field.jpg,’ and ‘Figure 1B-Mg-yellow field.jpg’ are original images of Mg mosquito line eye through blue, red, and yellow fluorescent filter, respectively; pictures of ‘Figure 1B-Sg-blue field.jpg,’ ‘Figure 1B-Sg-red field.jpg,’ and ‘-Figure 1B-Sg-yellow field.jpg’ are original images of Sg mosquito line eye through blue, red, and yellow fluorescent filter, respectively; pictures of ‘Figure 1B-E-blue field.jpg,’ ‘Figure 1B-E-red field.jpg,’ and ‘Figure 1B-E-yellow field.jpg’ are original images of E mosquito line eye through blue, red, and yellow fluorescent filter, respectively; pictures of Figure 1B-Mg-E-blue field.jpg, Figure 1B-Mg-E-red field.jpg, and Figure 1B-Mg-E-yellow field.jpg are original images of Mg/E mosquito line eye through blue, red, and yellow fluorescent filter, respectively; pictures of ‘Figure 1B-Sg-E-blue field.jpg,’ ‘Figure 1B-Sg-E-red field.jpg,’ and ‘Figure 1B-Sg-E-yellow field.jpg’ are original images of Sg/E mosquito line eye through blue, red, and yellow fluorescent filter, respectively; pictures of ‘Figure 1B-Mg+Sg-E-blue field.jpg,’ ‘Figure 1B-Mg+Sg-E-red field,’ and ‘Figure 1B-Mg+Sg-E-yellow field’ are original images of Mg/Sg/E mosquito line eye through blue, red, and yellow fluorescent filter, respectively. ‘Figure 1C and D-source data-RT-PCR data.xlsx’ is the original data for Figure 1C and D; ‘Figure 1C and D-gene expression.pzf’ shows Figure 1C and D were generated with GraphPad Prism. Pictures of ‘Figure 1E-western blot-MP2 in midgut.tif,’ ‘Figure 1E-western blot-Scorpine in midgut.tif,’ and ‘Figure 1E-western blot-α-tubulin in midgut.tif’ are original image of Western blots detected with mouse anti-MP2, mouse anti-scorpine, and rabbit anti-α-tubulin antibody. P [file elife-77584-fig1-data1.zip › Fig 1-source data/Fig1B-Mg-E Line-blue field.jpg]

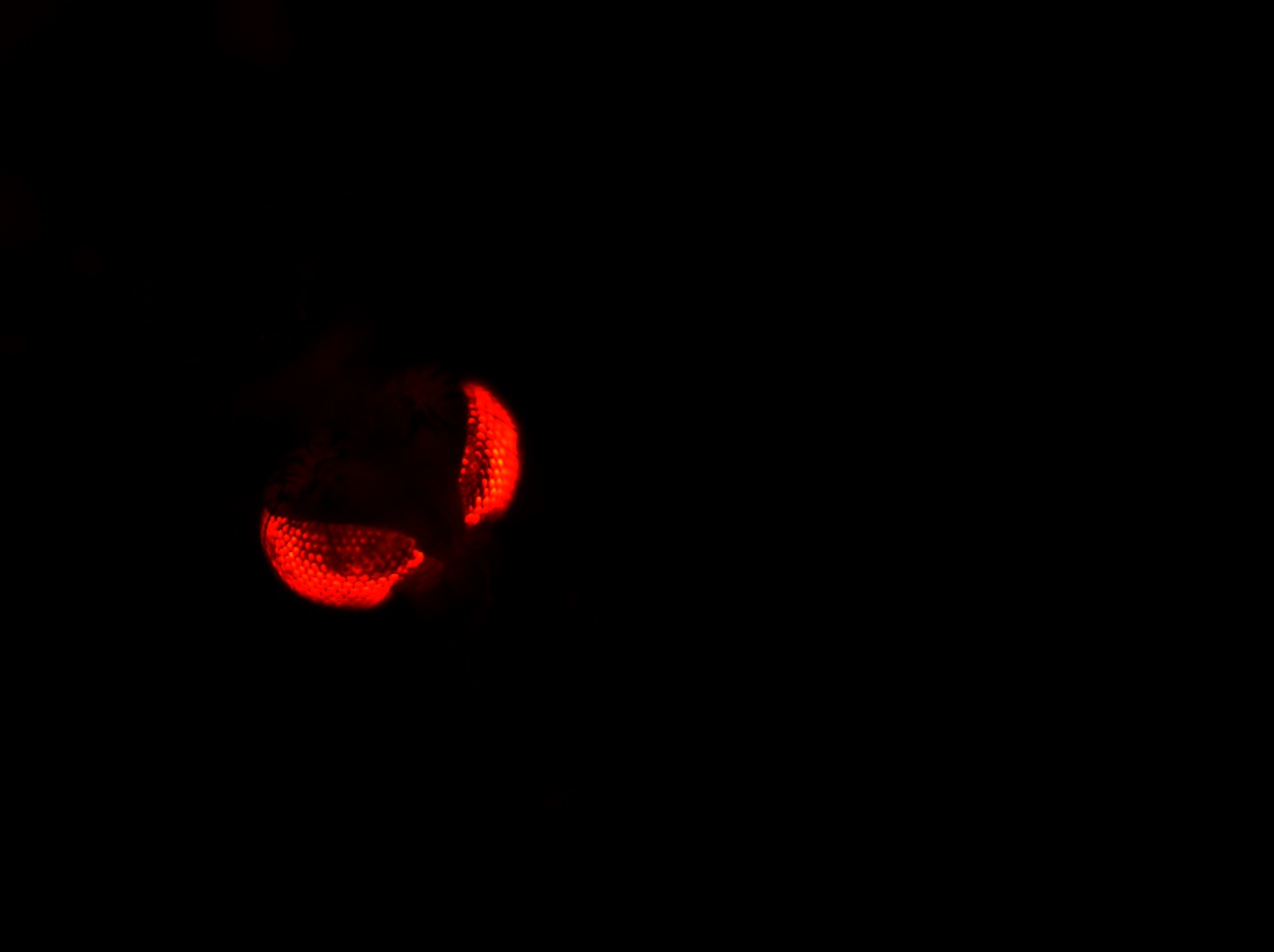

Supplement: Figure 1—source data 1. — Pictures of ‘Figure 1B-WT-blue field.tif,’ ‘Figure 1B-WT-red field.tif,’ and ‘Figure 1B-WT-yellow field.tif’ are original images of WT mosquito eye through blue, red, and yellow fluorescent filter, respectively; pictures of ‘Figure 1B-Mg-blue field.jpg,’ ‘Figure 1B-Mg-red field.jpg,’ and ‘Figure 1B-Mg-yellow field.jpg’ are original images of Mg mosquito line eye through blue, red, and yellow fluorescent filter, respectively; pictures of ‘Figure 1B-Sg-blue field.jpg,’ ‘Figure 1B-Sg-red field.jpg,’ and ‘-Figure 1B-Sg-yellow field.jpg’ are original images of Sg mosquito line eye through blue, red, and yellow fluorescent filter, respectively; pictures of ‘Figure 1B-E-blue field.jpg,’ ‘Figure 1B-E-red field.jpg,’ and ‘Figure 1B-E-yellow field.jpg’ are original images of E mosquito line eye through blue, red, and yellow fluorescent filter, respectively; pictures of Figure 1B-Mg-E-blue field.jpg, Figure 1B-Mg-E-red field.jpg, and Figure 1B-Mg-E-yellow field.jpg are original images of Mg/E mosquito line eye through blue, red, and yellow fluorescent filter, respectively; pictures of ‘Figure 1B-Sg-E-blue field.jpg,’ ‘Figure 1B-Sg-E-red field.jpg,’ and ‘Figure 1B-Sg-E-yellow field.jpg’ are original images of Sg/E mosquito line eye through blue, red, and yellow fluorescent filter, respectively; pictures of ‘Figure 1B-Mg+Sg-E-blue field.jpg,’ ‘Figure 1B-Mg+Sg-E-red field,’ and ‘Figure 1B-Mg+Sg-E-yellow field’ are original images of Mg/Sg/E mosquito line eye through blue, red, and yellow fluorescent filter, respectively. ‘Figure 1C and D-source data-RT-PCR data.xlsx’ is the original data for Figure 1C and D; ‘Figure 1C and D-gene expression.pzf’ shows Figure 1C and D were generated with GraphPad Prism. Pictures of ‘Figure 1E-western blot-MP2 in midgut.tif,’ ‘Figure 1E-western blot-Scorpine in midgut.tif,’ and ‘Figure 1E-western blot-α-tubulin in midgut.tif’ are original image of Western blots detected with mouse anti-MP2, mouse anti-scorpine, and rabbit anti-α-tubulin antibody. P [file elife-77584-fig1-data1.zip › Fig 1-source data/Fig1B-Mg-E Line-red field.jpg]

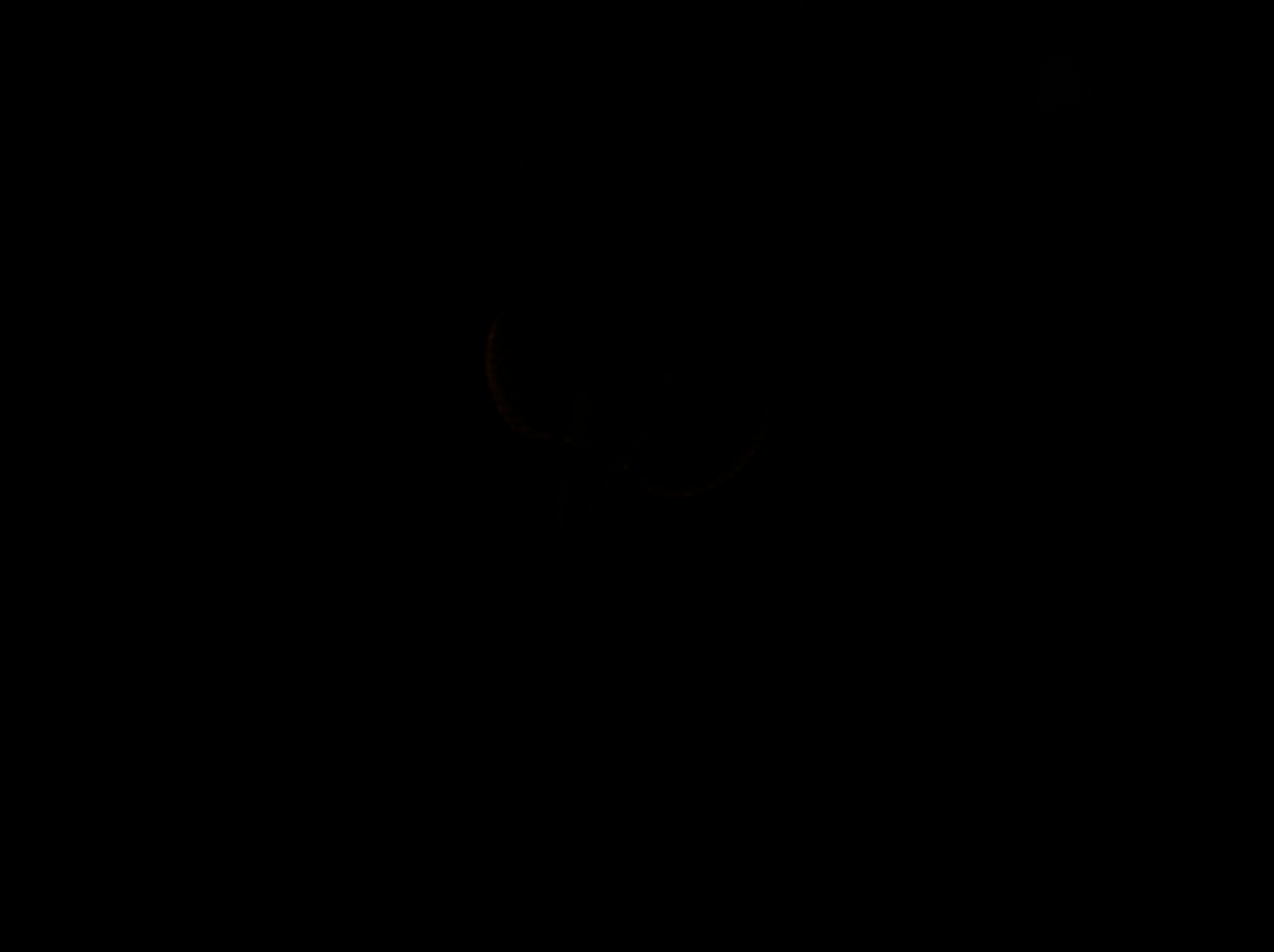

Supplement: Figure 1—source data 1. — Pictures of ‘Figure 1B-WT-blue field.tif,’ ‘Figure 1B-WT-red field.tif,’ and ‘Figure 1B-WT-yellow field.tif’ are original images of WT mosquito eye through blue, red, and yellow fluorescent filter, respectively; pictures of ‘Figure 1B-Mg-blue field.jpg,’ ‘Figure 1B-Mg-red field.jpg,’ and ‘Figure 1B-Mg-yellow field.jpg’ are original images of Mg mosquito line eye through blue, red, and yellow fluorescent filter, respectively; pictures of ‘Figure 1B-Sg-blue field.jpg,’ ‘Figure 1B-Sg-red field.jpg,’ and ‘-Figure 1B-Sg-yellow field.jpg’ are original images of Sg mosquito line eye through blue, red, and yellow fluorescent filter, respectively; pictures of ‘Figure 1B-E-blue field.jpg,’ ‘Figure 1B-E-red field.jpg,’ and ‘Figure 1B-E-yellow field.jpg’ are original images of E mosquito line eye through blue, red, and yellow fluorescent filter, respectively; pictures of Figure 1B-Mg-E-blue field.jpg, Figure 1B-Mg-E-red field.jpg, and Figure 1B-Mg-E-yellow field.jpg are original images of Mg/E mosquito line eye through blue, red, and yellow fluorescent filter, respectively; pictures of ‘Figure 1B-Sg-E-blue field.jpg,’ ‘Figure 1B-Sg-E-red field.jpg,’ and ‘Figure 1B-Sg-E-yellow field.jpg’ are original images of Sg/E mosquito line eye through blue, red, and yellow fluorescent filter, respectively; pictures of ‘Figure 1B-Mg+Sg-E-blue field.jpg,’ ‘Figure 1B-Mg+Sg-E-red field,’ and ‘Figure 1B-Mg+Sg-E-yellow field’ are original images of Mg/Sg/E mosquito line eye through blue, red, and yellow fluorescent filter, respectively. ‘Figure 1C and D-source data-RT-PCR data.xlsx’ is the original data for Figure 1C and D; ‘Figure 1C and D-gene expression.pzf’ shows Figure 1C and D were generated with GraphPad Prism. Pictures of ‘Figure 1E-western blot-MP2 in midgut.tif,’ ‘Figure 1E-western blot-Scorpine in midgut.tif,’ and ‘Figure 1E-western blot-α-tubulin in midgut.tif’ are original image of Western blots detected with mouse anti-MP2, mouse anti-scorpine, and rabbit anti-α-tubulin antibody. P [file elife-77584-fig1-data1.zip › Fig 1-source data/Fig1B-Mg line-yellow field.jpg]

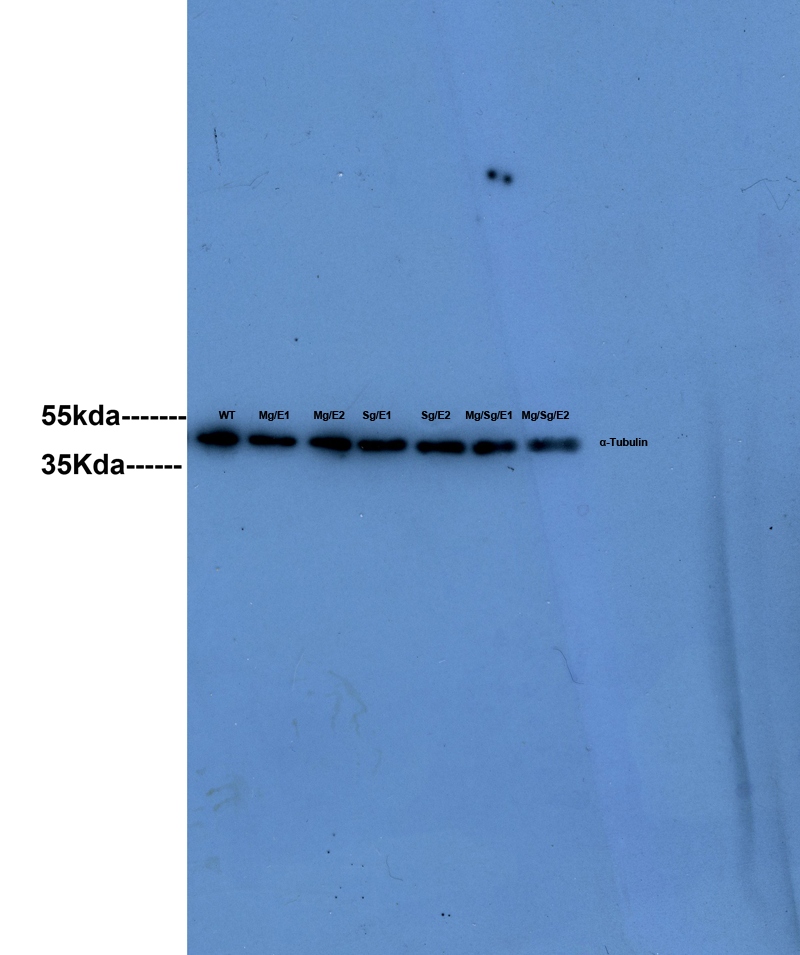

Supplement: Figure 1—source data 1. — Pictures of ‘Figure 1B-WT-blue field.tif,’ ‘Figure 1B-WT-red field.tif,’ and ‘Figure 1B-WT-yellow field.tif’ are original images of WT mosquito eye through blue, red, and yellow fluorescent filter, respectively; pictures of ‘Figure 1B-Mg-blue field.jpg,’ ‘Figure 1B-Mg-red field.jpg,’ and ‘Figure 1B-Mg-yellow field.jpg’ are original images of Mg mosquito line eye through blue, red, and yellow fluorescent filter, respectively; pictures of ‘Figure 1B-Sg-blue field.jpg,’ ‘Figure 1B-Sg-red field.jpg,’ and ‘-Figure 1B-Sg-yellow field.jpg’ are original images of Sg mosquito line eye through blue, red, and yellow fluorescent filter, respectively; pictures of ‘Figure 1B-E-blue field.jpg,’ ‘Figure 1B-E-red field.jpg,’ and ‘Figure 1B-E-yellow field.jpg’ are original images of E mosquito line eye through blue, red, and yellow fluorescent filter, respectively; pictures of Figure 1B-Mg-E-blue field.jpg, Figure 1B-Mg-E-red field.jpg, and Figure 1B-Mg-E-yellow field.jpg are original images of Mg/E mosquito line eye through blue, red, and yellow fluorescent filter, respectively; pictures of ‘Figure 1B-Sg-E-blue field.jpg,’ ‘Figure 1B-Sg-E-red field.jpg,’ and ‘Figure 1B-Sg-E-yellow field.jpg’ are original images of Sg/E mosquito line eye through blue, red, and yellow fluorescent filter, respectively; pictures of ‘Figure 1B-Mg+Sg-E-blue field.jpg,’ ‘Figure 1B-Mg+Sg-E-red field,’ and ‘Figure 1B-Mg+Sg-E-yellow field’ are original images of Mg/Sg/E mosquito line eye through blue, red, and yellow fluorescent filter, respectively. ‘Figure 1C and D-source data-RT-PCR data.xlsx’ is the original data for Figure 1C and D; ‘Figure 1C and D-gene expression.pzf’ shows Figure 1C and D were generated with GraphPad Prism. Pictures of ‘Figure 1E-western blot-MP2 in midgut.tif,’ ‘Figure 1E-western blot-Scorpine in midgut.tif,’ and ‘Figure 1E-western blot-α-tubulin in midgut.tif’ are original image of Western blots detected with mouse anti-MP2, mouse anti-scorpine, and rabbit anti-α-tubulin antibody. P [file elife-77584-fig1-data1.zip › Fig 1-source data/Fig 1F-western blot-a╠Ç-tubulin in salivary gland.tif]

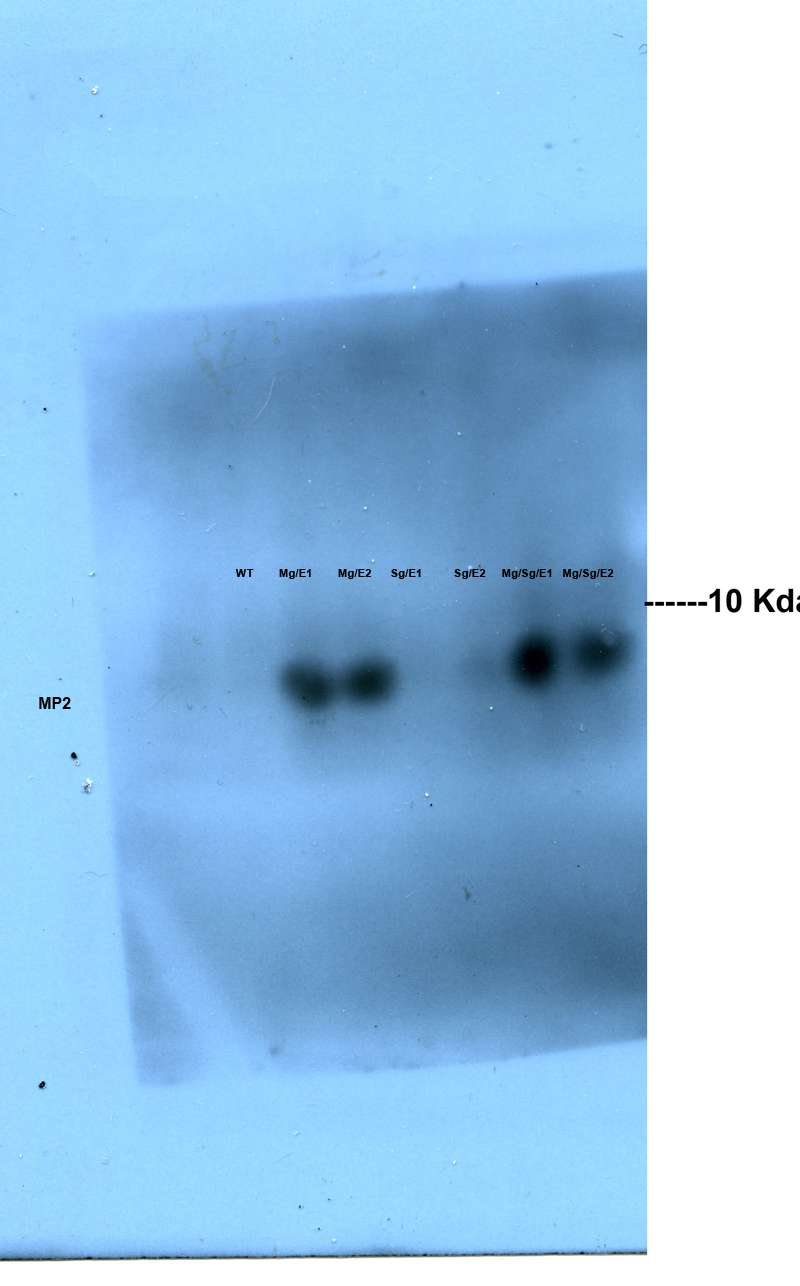

Supplement: Figure 1—source data 1. — Pictures of ‘Figure 1B-WT-blue field.tif,’ ‘Figure 1B-WT-red field.tif,’ and ‘Figure 1B-WT-yellow field.tif’ are original images of WT mosquito eye through blue, red, and yellow fluorescent filter, respectively; pictures of ‘Figure 1B-Mg-blue field.jpg,’ ‘Figure 1B-Mg-red field.jpg,’ and ‘Figure 1B-Mg-yellow field.jpg’ are original images of Mg mosquito line eye through blue, red, and yellow fluorescent filter, respectively; pictures of ‘Figure 1B-Sg-blue field.jpg,’ ‘Figure 1B-Sg-red field.jpg,’ and ‘-Figure 1B-Sg-yellow field.jpg’ are original images of Sg mosquito line eye through blue, red, and yellow fluorescent filter, respectively; pictures of ‘Figure 1B-E-blue field.jpg,’ ‘Figure 1B-E-red field.jpg,’ and ‘Figure 1B-E-yellow field.jpg’ are original images of E mosquito line eye through blue, red, and yellow fluorescent filter, respectively; pictures of Figure 1B-Mg-E-blue field.jpg, Figure 1B-Mg-E-red field.jpg, and Figure 1B-Mg-E-yellow field.jpg are original images of Mg/E mosquito line eye through blue, red, and yellow fluorescent filter, respectively; pictures of ‘Figure 1B-Sg-E-blue field.jpg,’ ‘Figure 1B-Sg-E-red field.jpg,’ and ‘Figure 1B-Sg-E-yellow field.jpg’ are original images of Sg/E mosquito line eye through blue, red, and yellow fluorescent filter, respectively; pictures of ‘Figure 1B-Mg+Sg-E-blue field.jpg,’ ‘Figure 1B-Mg+Sg-E-red field,’ and ‘Figure 1B-Mg+Sg-E-yellow field’ are original images of Mg/Sg/E mosquito line eye through blue, red, and yellow fluorescent filter, respectively. ‘Figure 1C and D-source data-RT-PCR data.xlsx’ is the original data for Figure 1C and D; ‘Figure 1C and D-gene expression.pzf’ shows Figure 1C and D were generated with GraphPad Prism. Pictures of ‘Figure 1E-western blot-MP2 in midgut.tif,’ ‘Figure 1E-western blot-Scorpine in midgut.tif,’ and ‘Figure 1E-western blot-α-tubulin in midgut.tif’ are original image of Western blots detected with mouse anti-MP2, mouse anti-scorpine, and rabbit anti-α-tubulin antibody. P [file elife-77584-fig1-data1.zip › Fig 1-source data/Fig 1E-western blot-MP2 in midgut.tif]

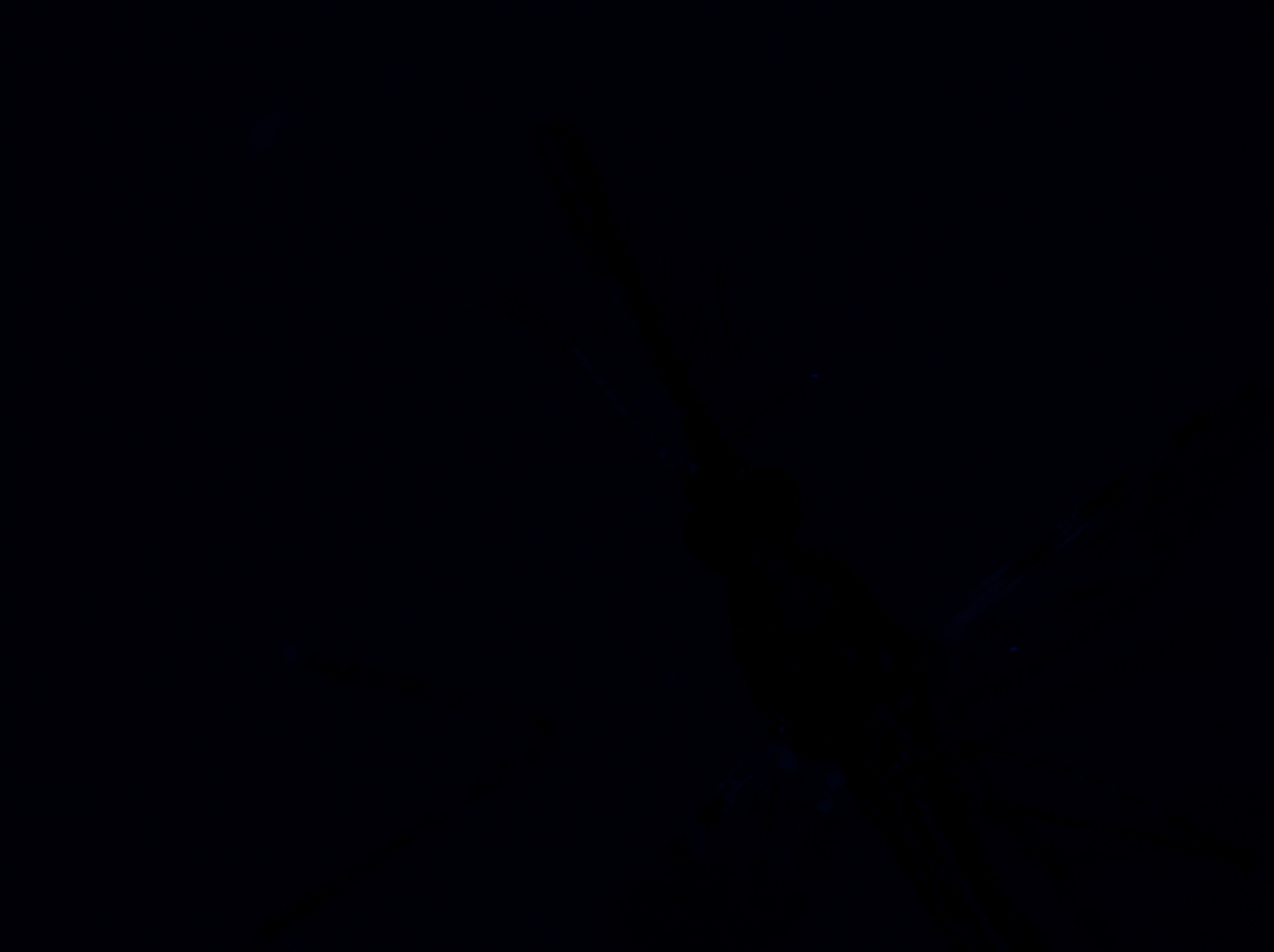

Supplement: Figure 1—source data 1. — Pictures of ‘Figure 1B-WT-blue field.tif,’ ‘Figure 1B-WT-red field.tif,’ and ‘Figure 1B-WT-yellow field.tif’ are original images of WT mosquito eye through blue, red, and yellow fluorescent filter, respectively; pictures of ‘Figure 1B-Mg-blue field.jpg,’ ‘Figure 1B-Mg-red field.jpg,’ and ‘Figure 1B-Mg-yellow field.jpg’ are original images of Mg mosquito line eye through blue, red, and yellow fluorescent filter, respectively; pictures of ‘Figure 1B-Sg-blue field.jpg,’ ‘Figure 1B-Sg-red field.jpg,’ and ‘-Figure 1B-Sg-yellow field.jpg’ are original images of Sg mosquito line eye through blue, red, and yellow fluorescent filter, respectively; pictures of ‘Figure 1B-E-blue field.jpg,’ ‘Figure 1B-E-red field.jpg,’ and ‘Figure 1B-E-yellow field.jpg’ are original images of E mosquito line eye through blue, red, and yellow fluorescent filter, respectively; pictures of Figure 1B-Mg-E-blue field.jpg, Figure 1B-Mg-E-red field.jpg, and Figure 1B-Mg-E-yellow field.jpg are original images of Mg/E mosquito line eye through blue, red, and yellow fluorescent filter, respectively; pictures of ‘Figure 1B-Sg-E-blue field.jpg,’ ‘Figure 1B-Sg-E-red field.jpg,’ and ‘Figure 1B-Sg-E-yellow field.jpg’ are original images of Sg/E mosquito line eye through blue, red, and yellow fluorescent filter, respectively; pictures of ‘Figure 1B-Mg+Sg-E-blue field.jpg,’ ‘Figure 1B-Mg+Sg-E-red field,’ and ‘Figure 1B-Mg+Sg-E-yellow field’ are original images of Mg/Sg/E mosquito line eye through blue, red, and yellow fluorescent filter, respectively. ‘Figure 1C and D-source data-RT-PCR data.xlsx’ is the original data for Figure 1C and D; ‘Figure 1C and D-gene expression.pzf’ shows Figure 1C and D were generated with GraphPad Prism. Pictures of ‘Figure 1E-western blot-MP2 in midgut.tif,’ ‘Figure 1E-western blot-Scorpine in midgut.tif,’ and ‘Figure 1E-western blot-α-tubulin in midgut.tif’ are original image of Western blots detected with mouse anti-MP2, mouse anti-scorpine, and rabbit anti-α-tubulin antibody. P [file elife-77584-fig1-data1.zip › Fig 1-source data/Fig1B-WT-blue field.tif]

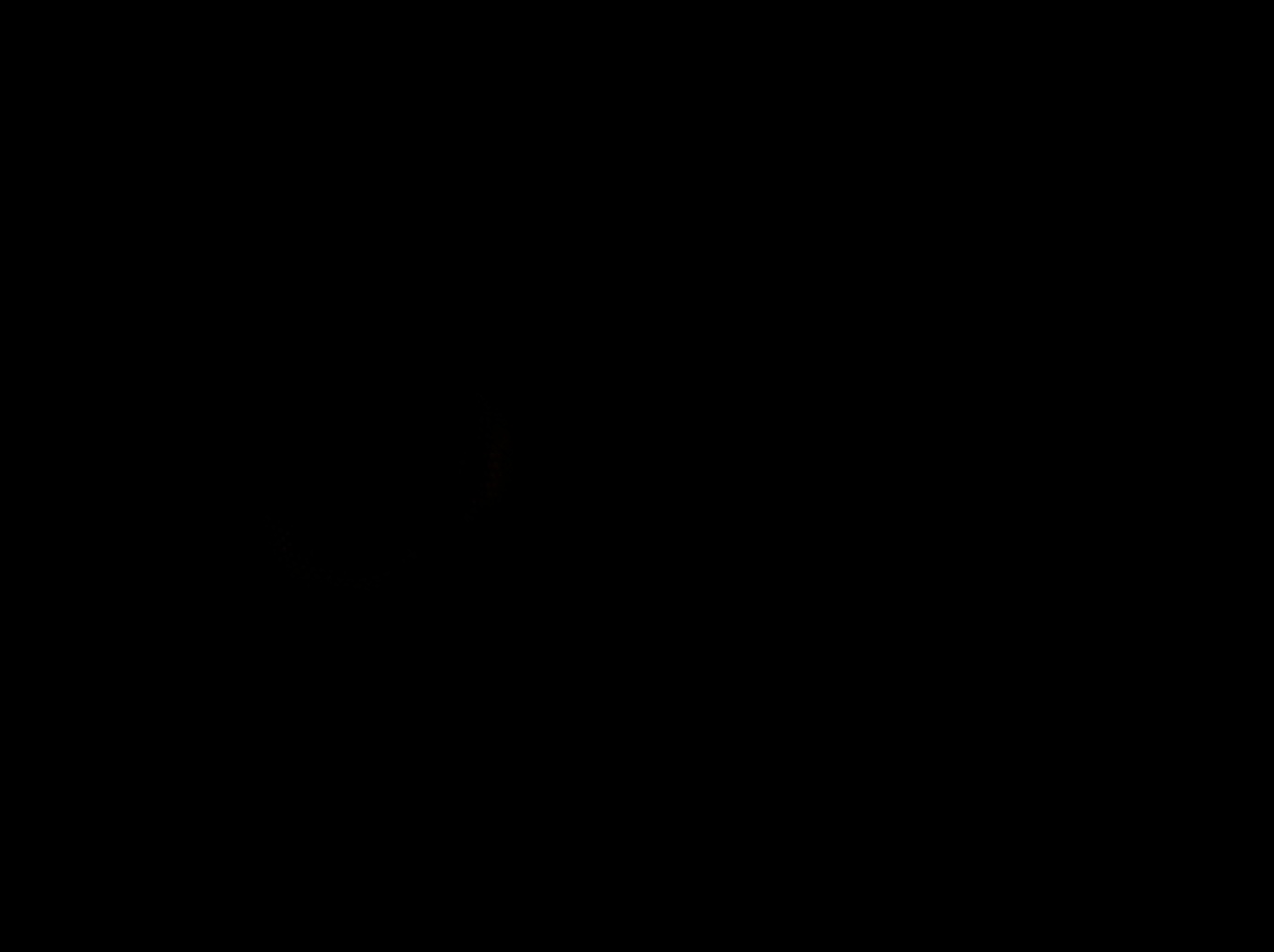

Supplement: Figure 1—source data 1. — Pictures of ‘Figure 1B-WT-blue field.tif,’ ‘Figure 1B-WT-red field.tif,’ and ‘Figure 1B-WT-yellow field.tif’ are original images of WT mosquito eye through blue, red, and yellow fluorescent filter, respectively; pictures of ‘Figure 1B-Mg-blue field.jpg,’ ‘Figure 1B-Mg-red field.jpg,’ and ‘Figure 1B-Mg-yellow field.jpg’ are original images of Mg mosquito line eye through blue, red, and yellow fluorescent filter, respectively; pictures of ‘Figure 1B-Sg-blue field.jpg,’ ‘Figure 1B-Sg-red field.jpg,’ and ‘-Figure 1B-Sg-yellow field.jpg’ are original images of Sg mosquito line eye through blue, red, and yellow fluorescent filter, respectively; pictures of ‘Figure 1B-E-blue field.jpg,’ ‘Figure 1B-E-red field.jpg,’ and ‘Figure 1B-E-yellow field.jpg’ are original images of E mosquito line eye through blue, red, and yellow fluorescent filter, respectively; pictures of Figure 1B-Mg-E-blue field.jpg, Figure 1B-Mg-E-red field.jpg, and Figure 1B-Mg-E-yellow field.jpg are original images of Mg/E mosquito line eye through blue, red, and yellow fluorescent filter, respectively; pictures of ‘Figure 1B-Sg-E-blue field.jpg,’ ‘Figure 1B-Sg-E-red field.jpg,’ and ‘Figure 1B-Sg-E-yellow field.jpg’ are original images of Sg/E mosquito line eye through blue, red, and yellow fluorescent filter, respectively; pictures of ‘Figure 1B-Mg+Sg-E-blue field.jpg,’ ‘Figure 1B-Mg+Sg-E-red field,’ and ‘Figure 1B-Mg+Sg-E-yellow field’ are original images of Mg/Sg/E mosquito line eye through blue, red, and yellow fluorescent filter, respectively. ‘Figure 1C and D-source data-RT-PCR data.xlsx’ is the original data for Figure 1C and D; ‘Figure 1C and D-gene expression.pzf’ shows Figure 1C and D were generated with GraphPad Prism. Pictures of ‘Figure 1E-western blot-MP2 in midgut.tif,’ ‘Figure 1E-western blot-Scorpine in midgut.tif,’ and ‘Figure 1E-western blot-α-tubulin in midgut.tif’ are original image of Western blots detected with mouse anti-MP2, mouse anti-scorpine, and rabbit anti-α-tubulin antibody. P [file elife-77584-fig1-data1.zip › Fig 1-source data/Fig1B-Mg-E Line-yellow field.jpg]

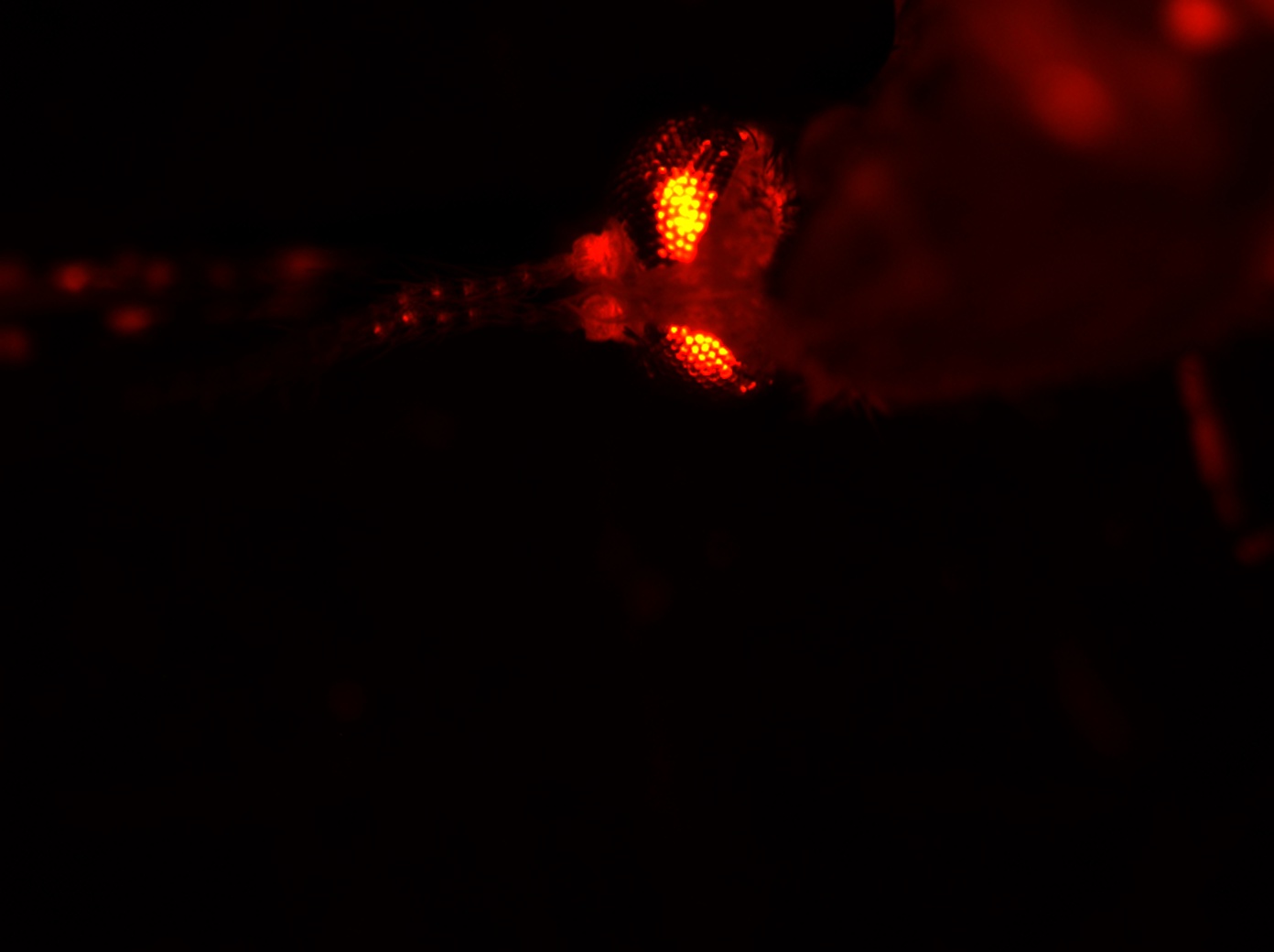

Supplement: Figure 1—source data 1. — Pictures of ‘Figure 1B-WT-blue field.tif,’ ‘Figure 1B-WT-red field.tif,’ and ‘Figure 1B-WT-yellow field.tif’ are original images of WT mosquito eye through blue, red, and yellow fluorescent filter, respectively; pictures of ‘Figure 1B-Mg-blue field.jpg,’ ‘Figure 1B-Mg-red field.jpg,’ and ‘Figure 1B-Mg-yellow field.jpg’ are original images of Mg mosquito line eye through blue, red, and yellow fluorescent filter, respectively; pictures of ‘Figure 1B-Sg-blue field.jpg,’ ‘Figure 1B-Sg-red field.jpg,’ and ‘-Figure 1B-Sg-yellow field.jpg’ are original images of Sg mosquito line eye through blue, red, and yellow fluorescent filter, respectively; pictures of ‘Figure 1B-E-blue field.jpg,’ ‘Figure 1B-E-red field.jpg,’ and ‘Figure 1B-E-yellow field.jpg’ are original images of E mosquito line eye through blue, red, and yellow fluorescent filter, respectively; pictures of Figure 1B-Mg-E-blue field.jpg, Figure 1B-Mg-E-red field.jpg, and Figure 1B-Mg-E-yellow field.jpg are original images of Mg/E mosquito line eye through blue, red, and yellow fluorescent filter, respectively; pictures of ‘Figure 1B-Sg-E-blue field.jpg,’ ‘Figure 1B-Sg-E-red field.jpg,’ and ‘Figure 1B-Sg-E-yellow field.jpg’ are original images of Sg/E mosquito line eye through blue, red, and yellow fluorescent filter, respectively; pictures of ‘Figure 1B-Mg+Sg-E-blue field.jpg,’ ‘Figure 1B-Mg+Sg-E-red field,’ and ‘Figure 1B-Mg+Sg-E-yellow field’ are original images of Mg/Sg/E mosquito line eye through blue, red, and yellow fluorescent filter, respectively. ‘Figure 1C and D-source data-RT-PCR data.xlsx’ is the original data for Figure 1C and D; ‘Figure 1C and D-gene expression.pzf’ shows Figure 1C and D were generated with GraphPad Prism. Pictures of ‘Figure 1E-western blot-MP2 in midgut.tif,’ ‘Figure 1E-western blot-Scorpine in midgut.tif,’ and ‘Figure 1E-western blot-α-tubulin in midgut.tif’ are original image of Western blots detected with mouse anti-MP2, mouse anti-scorpine, and rabbit anti-α-tubulin antibody. P [file elife-77584-fig1-data1.zip › Fig 1-source data/Fig1B-Mg+Sg-E line-red field.jpg]

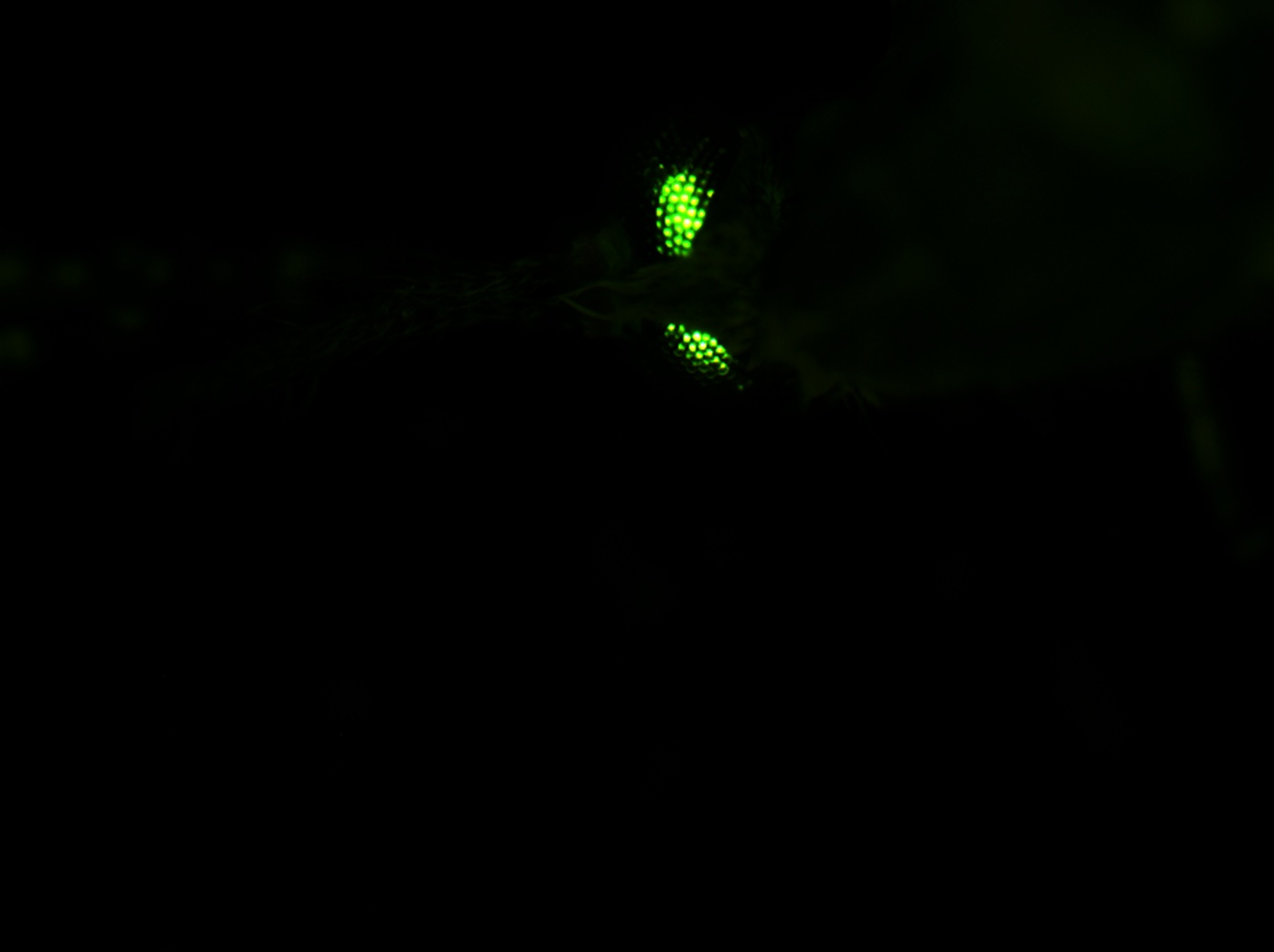

Supplement: Figure 1—source data 1. — Pictures of ‘Figure 1B-WT-blue field.tif,’ ‘Figure 1B-WT-red field.tif,’ and ‘Figure 1B-WT-yellow field.tif’ are original images of WT mosquito eye through blue, red, and yellow fluorescent filter, respectively; pictures of ‘Figure 1B-Mg-blue field.jpg,’ ‘Figure 1B-Mg-red field.jpg,’ and ‘Figure 1B-Mg-yellow field.jpg’ are original images of Mg mosquito line eye through blue, red, and yellow fluorescent filter, respectively; pictures of ‘Figure 1B-Sg-blue field.jpg,’ ‘Figure 1B-Sg-red field.jpg,’ and ‘-Figure 1B-Sg-yellow field.jpg’ are original images of Sg mosquito line eye through blue, red, and yellow fluorescent filter, respectively; pictures of ‘Figure 1B-E-blue field.jpg,’ ‘Figure 1B-E-red field.jpg,’ and ‘Figure 1B-E-yellow field.jpg’ are original images of E mosquito line eye through blue, red, and yellow fluorescent filter, respectively; pictures of Figure 1B-Mg-E-blue field.jpg, Figure 1B-Mg-E-red field.jpg, and Figure 1B-Mg-E-yellow field.jpg are original images of Mg/E mosquito line eye through blue, red, and yellow fluorescent filter, respectively; pictures of ‘Figure 1B-Sg-E-blue field.jpg,’ ‘Figure 1B-Sg-E-red field.jpg,’ and ‘Figure 1B-Sg-E-yellow field.jpg’ are original images of Sg/E mosquito line eye through blue, red, and yellow fluorescent filter, respectively; pictures of ‘Figure 1B-Mg+Sg-E-blue field.jpg,’ ‘Figure 1B-Mg+Sg-E-red field,’ and ‘Figure 1B-Mg+Sg-E-yellow field’ are original images of Mg/Sg/E mosquito line eye through blue, red, and yellow fluorescent filter, respectively. ‘Figure 1C and D-source data-RT-PCR data.xlsx’ is the original data for Figure 1C and D; ‘Figure 1C and D-gene expression.pzf’ shows Figure 1C and D were generated with GraphPad Prism. Pictures of ‘Figure 1E-western blot-MP2 in midgut.tif,’ ‘Figure 1E-western blot-Scorpine in midgut.tif,’ and ‘Figure 1E-western blot-α-tubulin in midgut.tif’ are original image of Western blots detected with mouse anti-MP2, mouse anti-scorpine, and rabbit anti-α-tubulin antibody. P [file elife-77584-fig1-data1.zip › Fig 1-source data/Fig1B-Mg+Sg-E line-yellow field.jpg]

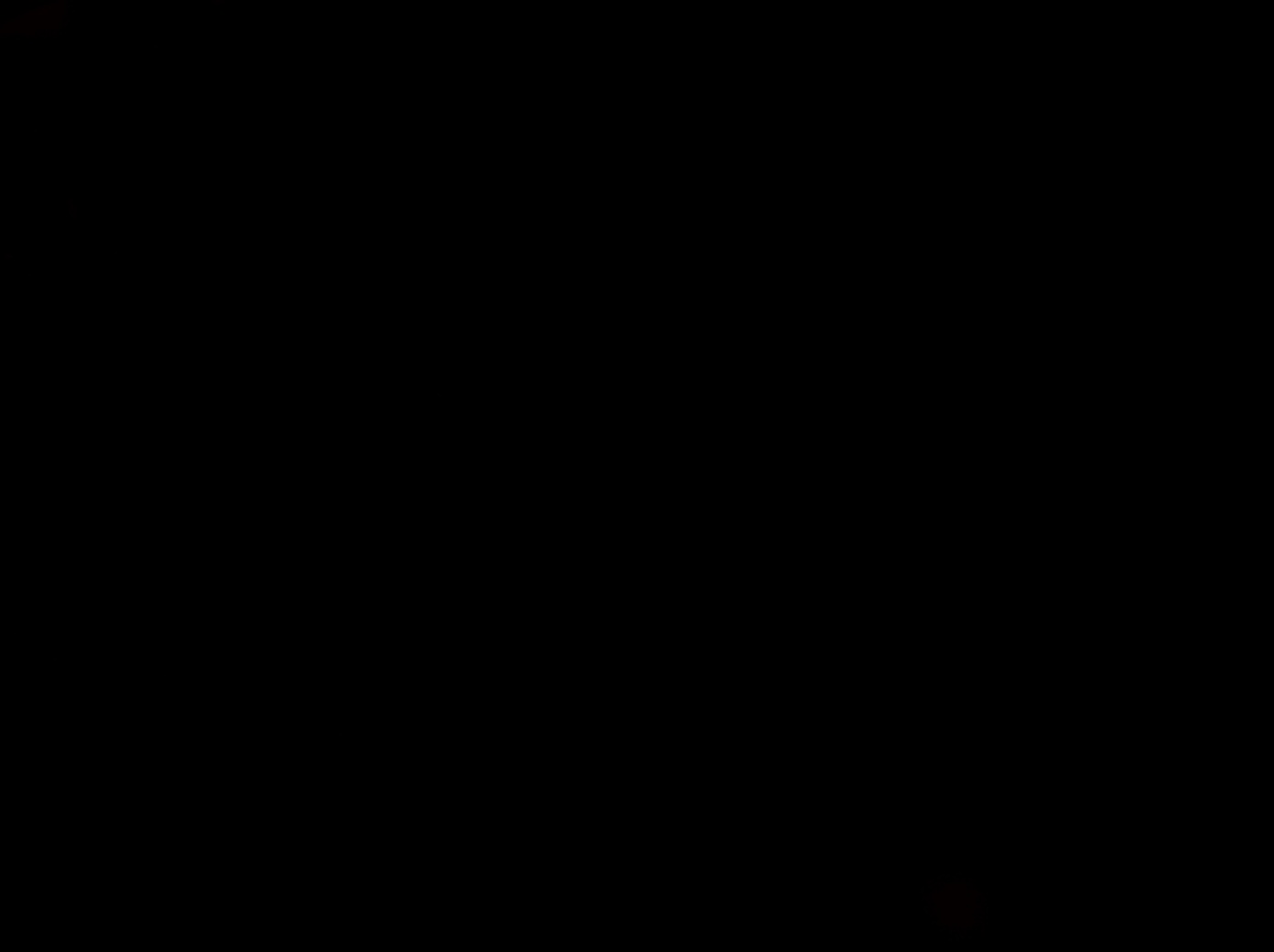

Supplement: Figure 1—source data 1. — Pictures of ‘Figure 1B-WT-blue field.tif,’ ‘Figure 1B-WT-red field.tif,’ and ‘Figure 1B-WT-yellow field.tif’ are original images of WT mosquito eye through blue, red, and yellow fluorescent filter, respectively; pictures of ‘Figure 1B-Mg-blue field.jpg,’ ‘Figure 1B-Mg-red field.jpg,’ and ‘Figure 1B-Mg-yellow field.jpg’ are original images of Mg mosquito line eye through blue, red, and yellow fluorescent filter, respectively; pictures of ‘Figure 1B-Sg-blue field.jpg,’ ‘Figure 1B-Sg-red field.jpg,’ and ‘-Figure 1B-Sg-yellow field.jpg’ are original images of Sg mosquito line eye through blue, red, and yellow fluorescent filter, respectively; pictures of ‘Figure 1B-E-blue field.jpg,’ ‘Figure 1B-E-red field.jpg,’ and ‘Figure 1B-E-yellow field.jpg’ are original images of E mosquito line eye through blue, red, and yellow fluorescent filter, respectively; pictures of Figure 1B-Mg-E-blue field.jpg, Figure 1B-Mg-E-red field.jpg, and Figure 1B-Mg-E-yellow field.jpg are original images of Mg/E mosquito line eye through blue, red, and yellow fluorescent filter, respectively; pictures of ‘Figure 1B-Sg-E-blue field.jpg,’ ‘Figure 1B-Sg-E-red field.jpg,’ and ‘Figure 1B-Sg-E-yellow field.jpg’ are original images of Sg/E mosquito line eye through blue, red, and yellow fluorescent filter, respectively; pictures of ‘Figure 1B-Mg+Sg-E-blue field.jpg,’ ‘Figure 1B-Mg+Sg-E-red field,’ and ‘Figure 1B-Mg+Sg-E-yellow field’ are original images of Mg/Sg/E mosquito line eye through blue, red, and yellow fluorescent filter, respectively. ‘Figure 1C and D-source data-RT-PCR data.xlsx’ is the original data for Figure 1C and D; ‘Figure 1C and D-gene expression.pzf’ shows Figure 1C and D were generated with GraphPad Prism. Pictures of ‘Figure 1E-western blot-MP2 in midgut.tif,’ ‘Figure 1E-western blot-Scorpine in midgut.tif,’ and ‘Figure 1E-western blot-α-tubulin in midgut.tif’ are original image of Western blots detected with mouse anti-MP2, mouse anti-scorpine, and rabbit anti-α-tubulin antibody. P [file elife-77584-fig1-data1.zip › Fig 1-source data/Fig1B-E line-red filed.jpg]

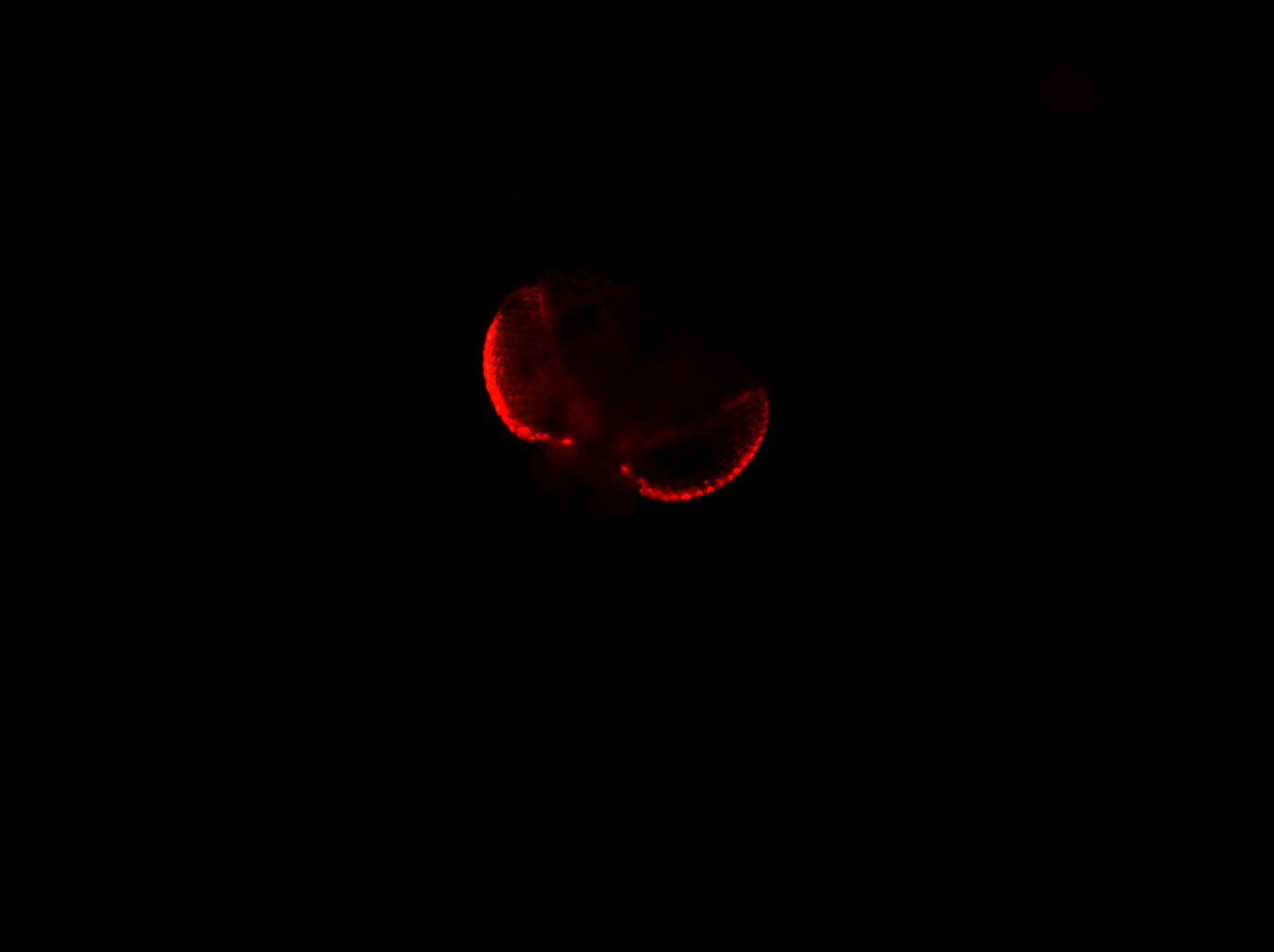

Supplement: Figure 1—source data 1. — Pictures of ‘Figure 1B-WT-blue field.tif,’ ‘Figure 1B-WT-red field.tif,’ and ‘Figure 1B-WT-yellow field.tif’ are original images of WT mosquito eye through blue, red, and yellow fluorescent filter, respectively; pictures of ‘Figure 1B-Mg-blue field.jpg,’ ‘Figure 1B-Mg-red field.jpg,’ and ‘Figure 1B-Mg-yellow field.jpg’ are original images of Mg mosquito line eye through blue, red, and yellow fluorescent filter, respectively; pictures of ‘Figure 1B-Sg-blue field.jpg,’ ‘Figure 1B-Sg-red field.jpg,’ and ‘-Figure 1B-Sg-yellow field.jpg’ are original images of Sg mosquito line eye through blue, red, and yellow fluorescent filter, respectively; pictures of ‘Figure 1B-E-blue field.jpg,’ ‘Figure 1B-E-red field.jpg,’ and ‘Figure 1B-E-yellow field.jpg’ are original images of E mosquito line eye through blue, red, and yellow fluorescent filter, respectively; pictures of Figure 1B-Mg-E-blue field.jpg, Figure 1B-Mg-E-red field.jpg, and Figure 1B-Mg-E-yellow field.jpg are original images of Mg/E mosquito line eye through blue, red, and yellow fluorescent filter, respectively; pictures of ‘Figure 1B-Sg-E-blue field.jpg,’ ‘Figure 1B-Sg-E-red field.jpg,’ and ‘Figure 1B-Sg-E-yellow field.jpg’ are original images of Sg/E mosquito line eye through blue, red, and yellow fluorescent filter, respectively; pictures of ‘Figure 1B-Mg+Sg-E-blue field.jpg,’ ‘Figure 1B-Mg+Sg-E-red field,’ and ‘Figure 1B-Mg+Sg-E-yellow field’ are original images of Mg/Sg/E mosquito line eye through blue, red, and yellow fluorescent filter, respectively. ‘Figure 1C and D-source data-RT-PCR data.xlsx’ is the original data for Figure 1C and D; ‘Figure 1C and D-gene expression.pzf’ shows Figure 1C and D were generated with GraphPad Prism. Pictures of ‘Figure 1E-western blot-MP2 in midgut.tif,’ ‘Figure 1E-western blot-Scorpine in midgut.tif,’ and ‘Figure 1E-western blot-α-tubulin in midgut.tif’ are original image of Western blots detected with mouse anti-MP2, mouse anti-scorpine, and rabbit anti-α-tubulin antibody. P [file elife-77584-fig1-data1.zip › Fig 1-source data/Fig1B-Mg line-red field.jpg]

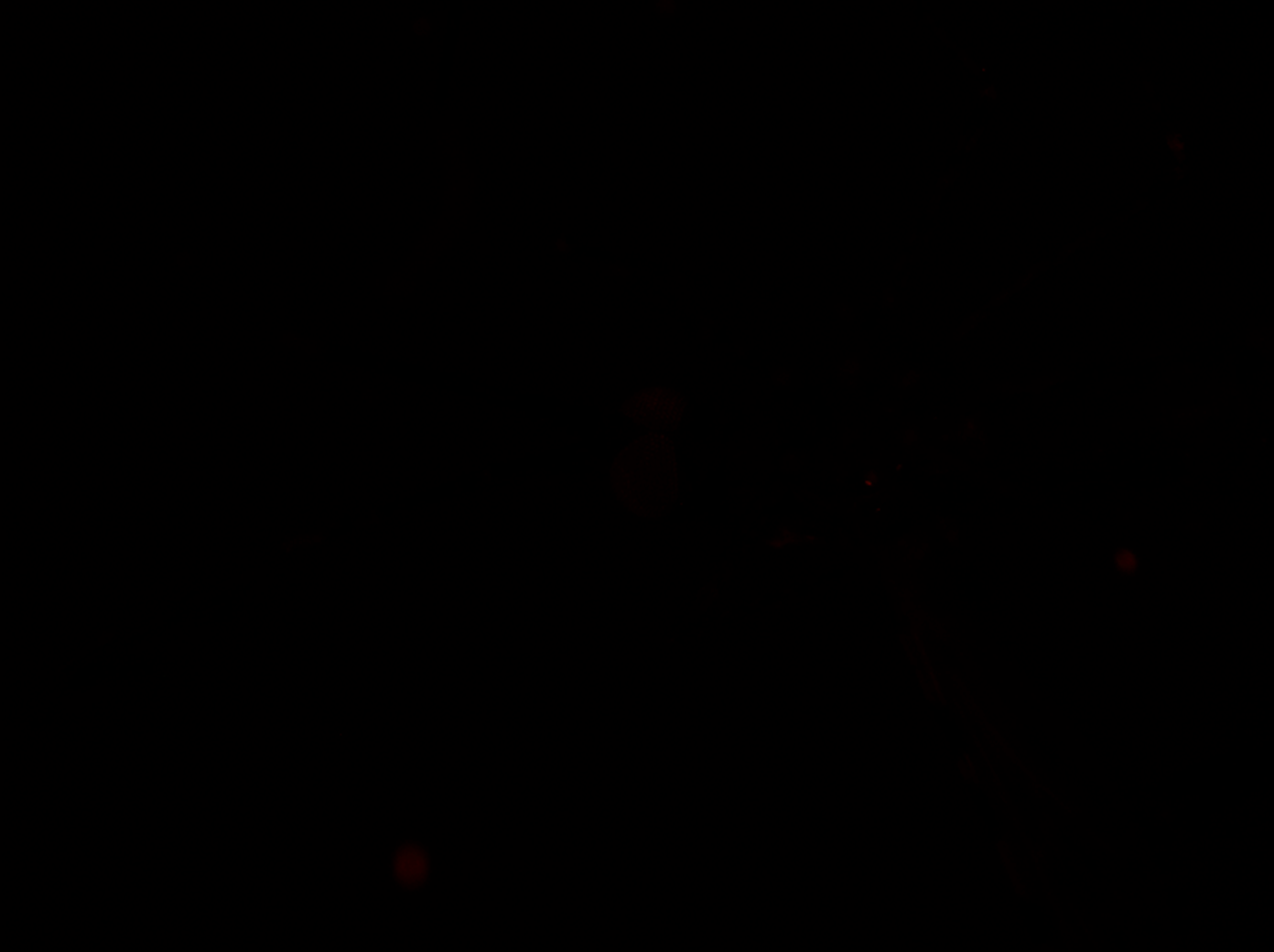

Supplement: Figure 1—source data 1. — Pictures of ‘Figure 1B-WT-blue field.tif,’ ‘Figure 1B-WT-red field.tif,’ and ‘Figure 1B-WT-yellow field.tif’ are original images of WT mosquito eye through blue, red, and yellow fluorescent filter, respectively; pictures of ‘Figure 1B-Mg-blue field.jpg,’ ‘Figure 1B-Mg-red field.jpg,’ and ‘Figure 1B-Mg-yellow field.jpg’ are original images of Mg mosquito line eye through blue, red, and yellow fluorescent filter, respectively; pictures of ‘Figure 1B-Sg-blue field.jpg,’ ‘Figure 1B-Sg-red field.jpg,’ and ‘-Figure 1B-Sg-yellow field.jpg’ are original images of Sg mosquito line eye through blue, red, and yellow fluorescent filter, respectively; pictures of ‘Figure 1B-E-blue field.jpg,’ ‘Figure 1B-E-red field.jpg,’ and ‘Figure 1B-E-yellow field.jpg’ are original images of E mosquito line eye through blue, red, and yellow fluorescent filter, respectively; pictures of Figure 1B-Mg-E-blue field.jpg, Figure 1B-Mg-E-red field.jpg, and Figure 1B-Mg-E-yellow field.jpg are original images of Mg/E mosquito line eye through blue, red, and yellow fluorescent filter, respectively; pictures of ‘Figure 1B-Sg-E-blue field.jpg,’ ‘Figure 1B-Sg-E-red field.jpg,’ and ‘Figure 1B-Sg-E-yellow field.jpg’ are original images of Sg/E mosquito line eye through blue, red, and yellow fluorescent filter, respectively; pictures of ‘Figure 1B-Mg+Sg-E-blue field.jpg,’ ‘Figure 1B-Mg+Sg-E-red field,’ and ‘Figure 1B-Mg+Sg-E-yellow field’ are original images of Mg/Sg/E mosquito line eye through blue, red, and yellow fluorescent filter, respectively. ‘Figure 1C and D-source data-RT-PCR data.xlsx’ is the original data for Figure 1C and D; ‘Figure 1C and D-gene expression.pzf’ shows Figure 1C and D were generated with GraphPad Prism. Pictures of ‘Figure 1E-western blot-MP2 in midgut.tif,’ ‘Figure 1E-western blot-Scorpine in midgut.tif,’ and ‘Figure 1E-western blot-α-tubulin in midgut.tif’ are original image of Western blots detected with mouse anti-MP2, mouse anti-scorpine, and rabbit anti-α-tubulin antibody. P [file elife-77584-fig1-data1.zip › Fig 1-source data/Fig1B-Sg line-red field.tif]

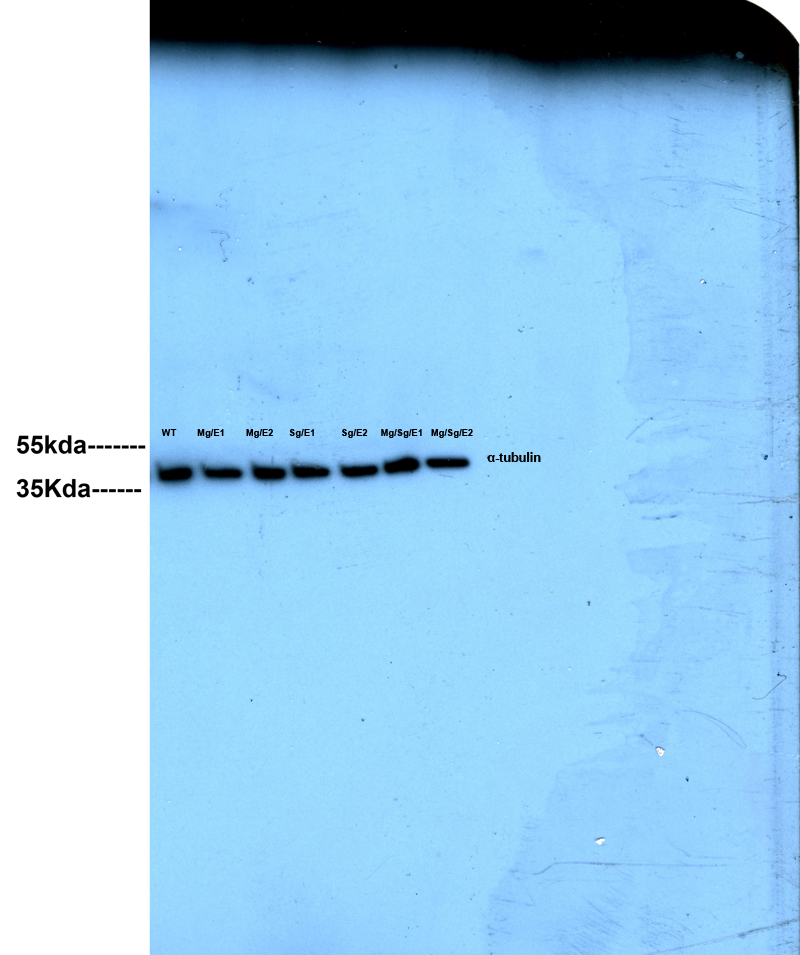

Supplement: Figure 1—source data 1. — Pictures of ‘Figure 1B-WT-blue field.tif,’ ‘Figure 1B-WT-red field.tif,’ and ‘Figure 1B-WT-yellow field.tif’ are original images of WT mosquito eye through blue, red, and yellow fluorescent filter, respectively; pictures of ‘Figure 1B-Mg-blue field.jpg,’ ‘Figure 1B-Mg-red field.jpg,’ and ‘Figure 1B-Mg-yellow field.jpg’ are original images of Mg mosquito line eye through blue, red, and yellow fluorescent filter, respectively; pictures of ‘Figure 1B-Sg-blue field.jpg,’ ‘Figure 1B-Sg-red field.jpg,’ and ‘-Figure 1B-Sg-yellow field.jpg’ are original images of Sg mosquito line eye through blue, red, and yellow fluorescent filter, respectively; pictures of ‘Figure 1B-E-blue field.jpg,’ ‘Figure 1B-E-red field.jpg,’ and ‘Figure 1B-E-yellow field.jpg’ are original images of E mosquito line eye through blue, red, and yellow fluorescent filter, respectively; pictures of Figure 1B-Mg-E-blue field.jpg, Figure 1B-Mg-E-red field.jpg, and Figure 1B-Mg-E-yellow field.jpg are original images of Mg/E mosquito line eye through blue, red, and yellow fluorescent filter, respectively; pictures of ‘Figure 1B-Sg-E-blue field.jpg,’ ‘Figure 1B-Sg-E-red field.jpg,’ and ‘Figure 1B-Sg-E-yellow field.jpg’ are original images of Sg/E mosquito line eye through blue, red, and yellow fluorescent filter, respectively; pictures of ‘Figure 1B-Mg+Sg-E-blue field.jpg,’ ‘Figure 1B-Mg+Sg-E-red field,’ and ‘Figure 1B-Mg+Sg-E-yellow field’ are original images of Mg/Sg/E mosquito line eye through blue, red, and yellow fluorescent filter, respectively. ‘Figure 1C and D-source data-RT-PCR data.xlsx’ is the original data for Figure 1C and D; ‘Figure 1C and D-gene expression.pzf’ shows Figure 1C and D were generated with GraphPad Prism. Pictures of ‘Figure 1E-western blot-MP2 in midgut.tif,’ ‘Figure 1E-western blot-Scorpine in midgut.tif,’ and ‘Figure 1E-western blot-α-tubulin in midgut.tif’ are original image of Western blots detected with mouse anti-MP2, mouse anti-scorpine, and rabbit anti-α-tubulin antibody. P [file elife-77584-fig1-data1.zip › Fig 1-source data/Fig1E-western blot- a╠Ç-tubulin in midgut.tif]

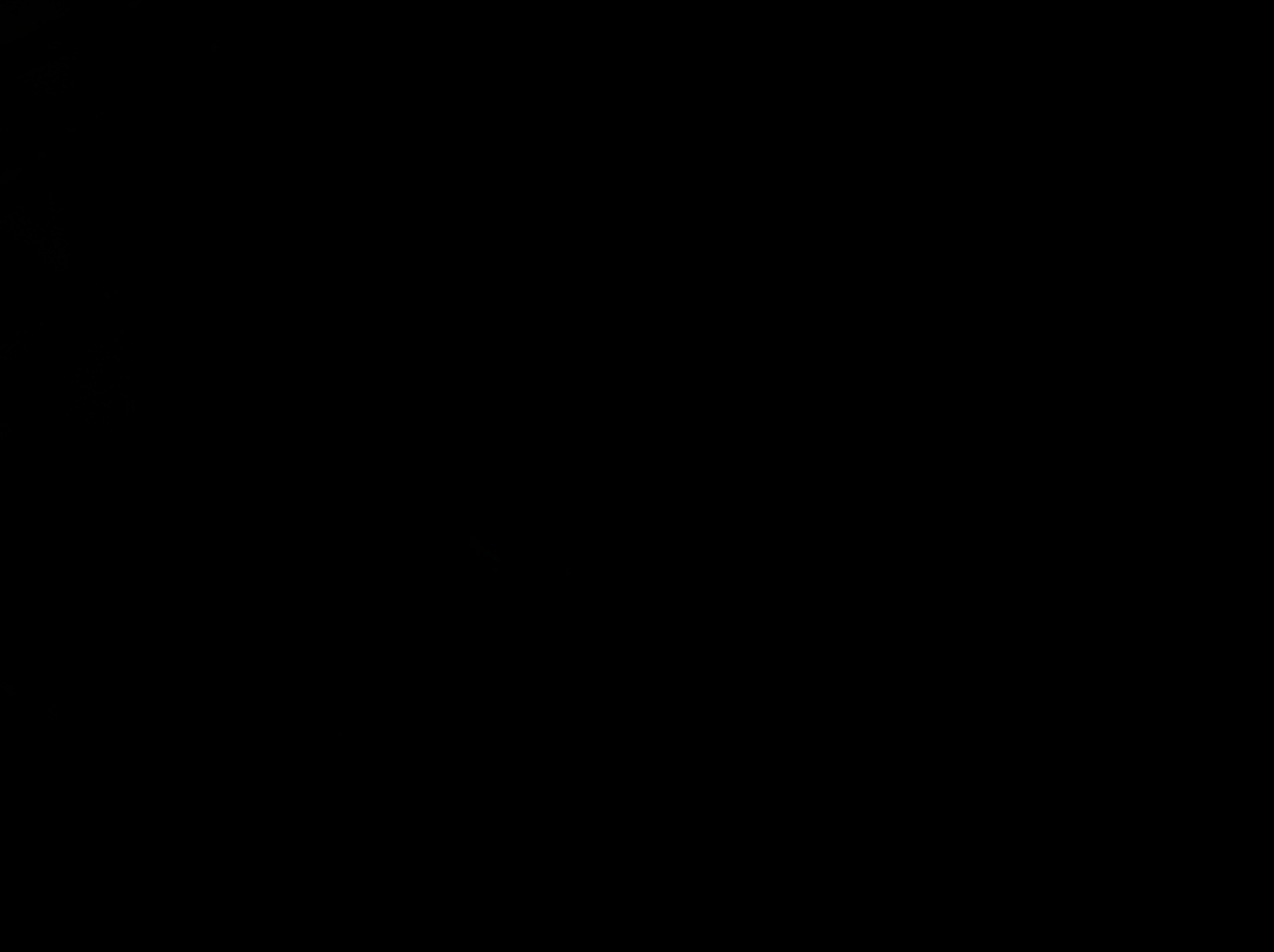

Supplement: Figure 1—source data 1. — Pictures of ‘Figure 1B-WT-blue field.tif,’ ‘Figure 1B-WT-red field.tif,’ and ‘Figure 1B-WT-yellow field.tif’ are original images of WT mosquito eye through blue, red, and yellow fluorescent filter, respectively; pictures of ‘Figure 1B-Mg-blue field.jpg,’ ‘Figure 1B-Mg-red field.jpg,’ and ‘Figure 1B-Mg-yellow field.jpg’ are original images of Mg mosquito line eye through blue, red, and yellow fluorescent filter, respectively; pictures of ‘Figure 1B-Sg-blue field.jpg,’ ‘Figure 1B-Sg-red field.jpg,’ and ‘-Figure 1B-Sg-yellow field.jpg’ are original images of Sg mosquito line eye through blue, red, and yellow fluorescent filter, respectively; pictures of ‘Figure 1B-E-blue field.jpg,’ ‘Figure 1B-E-red field.jpg,’ and ‘Figure 1B-E-yellow field.jpg’ are original images of E mosquito line eye through blue, red, and yellow fluorescent filter, respectively; pictures of Figure 1B-Mg-E-blue field.jpg, Figure 1B-Mg-E-red field.jpg, and Figure 1B-Mg-E-yellow field.jpg are original images of Mg/E mosquito line eye through blue, red, and yellow fluorescent filter, respectively; pictures of ‘Figure 1B-Sg-E-blue field.jpg,’ ‘Figure 1B-Sg-E-red field.jpg,’ and ‘Figure 1B-Sg-E-yellow field.jpg’ are original images of Sg/E mosquito line eye through blue, red, and yellow fluorescent filter, respectively; pictures of ‘Figure 1B-Mg+Sg-E-blue field.jpg,’ ‘Figure 1B-Mg+Sg-E-red field,’ and ‘Figure 1B-Mg+Sg-E-yellow field’ are original images of Mg/Sg/E mosquito line eye through blue, red, and yellow fluorescent filter, respectively. ‘Figure 1C and D-source data-RT-PCR data.xlsx’ is the original data for Figure 1C and D; ‘Figure 1C and D-gene expression.pzf’ shows Figure 1C and D were generated with GraphPad Prism. Pictures of ‘Figure 1E-western blot-MP2 in midgut.tif,’ ‘Figure 1E-western blot-Scorpine in midgut.tif,’ and ‘Figure 1E-western blot-α-tubulin in midgut.tif’ are original image of Western blots detected with mouse anti-MP2, mouse anti-scorpine, and rabbit anti-α-tubulin antibody. P [file elife-77584-fig1-data1.zip › Fig 1-source data/Fig1B-E line-yellow filed.jpg]

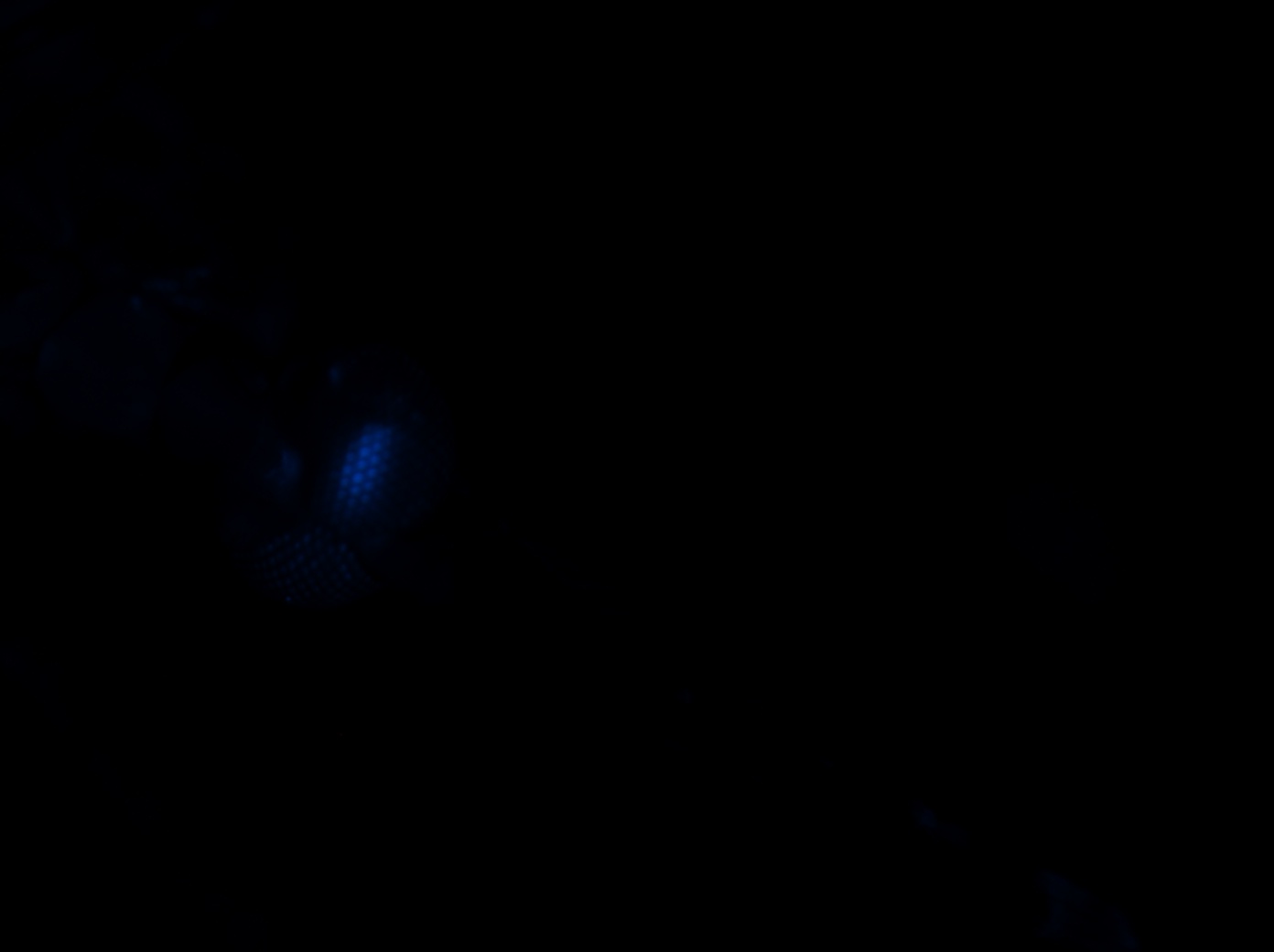

Supplement: Figure 1—source data 1. — Pictures of ‘Figure 1B-WT-blue field.tif,’ ‘Figure 1B-WT-red field.tif,’ and ‘Figure 1B-WT-yellow field.tif’ are original images of WT mosquito eye through blue, red, and yellow fluorescent filter, respectively; pictures of ‘Figure 1B-Mg-blue field.jpg,’ ‘Figure 1B-Mg-red field.jpg,’ and ‘Figure 1B-Mg-yellow field.jpg’ are original images of Mg mosquito line eye through blue, red, and yellow fluorescent filter, respectively; pictures of ‘Figure 1B-Sg-blue field.jpg,’ ‘Figure 1B-Sg-red field.jpg,’ and ‘-Figure 1B-Sg-yellow field.jpg’ are original images of Sg mosquito line eye through blue, red, and yellow fluorescent filter, respectively; pictures of ‘Figure 1B-E-blue field.jpg,’ ‘Figure 1B-E-red field.jpg,’ and ‘Figure 1B-E-yellow field.jpg’ are original images of E mosquito line eye through blue, red, and yellow fluorescent filter, respectively; pictures of Figure 1B-Mg-E-blue field.jpg, Figure 1B-Mg-E-red field.jpg, and Figure 1B-Mg-E-yellow field.jpg are original images of Mg/E mosquito line eye through blue, red, and yellow fluorescent filter, respectively; pictures of ‘Figure 1B-Sg-E-blue field.jpg,’ ‘Figure 1B-Sg-E-red field.jpg,’ and ‘Figure 1B-Sg-E-yellow field.jpg’ are original images of Sg/E mosquito line eye through blue, red, and yellow fluorescent filter, respectively; pictures of ‘Figure 1B-Mg+Sg-E-blue field.jpg,’ ‘Figure 1B-Mg+Sg-E-red field,’ and ‘Figure 1B-Mg+Sg-E-yellow field’ are original images of Mg/Sg/E mosquito line eye through blue, red, and yellow fluorescent filter, respectively. ‘Figure 1C and D-source data-RT-PCR data.xlsx’ is the original data for Figure 1C and D; ‘Figure 1C and D-gene expression.pzf’ shows Figure 1C and D were generated with GraphPad Prism. Pictures of ‘Figure 1E-western blot-MP2 in midgut.tif,’ ‘Figure 1E-western blot-Scorpine in midgut.tif,’ and ‘Figure 1E-western blot-α-tubulin in midgut.tif’ are original image of Western blots detected with mouse anti-MP2, mouse anti-scorpine, and rabbit anti-α-tubulin antibody. P [file elife-77584-fig1-data1.zip › Fig 1-source data/Fig1B-E line-blue filed.jpg]

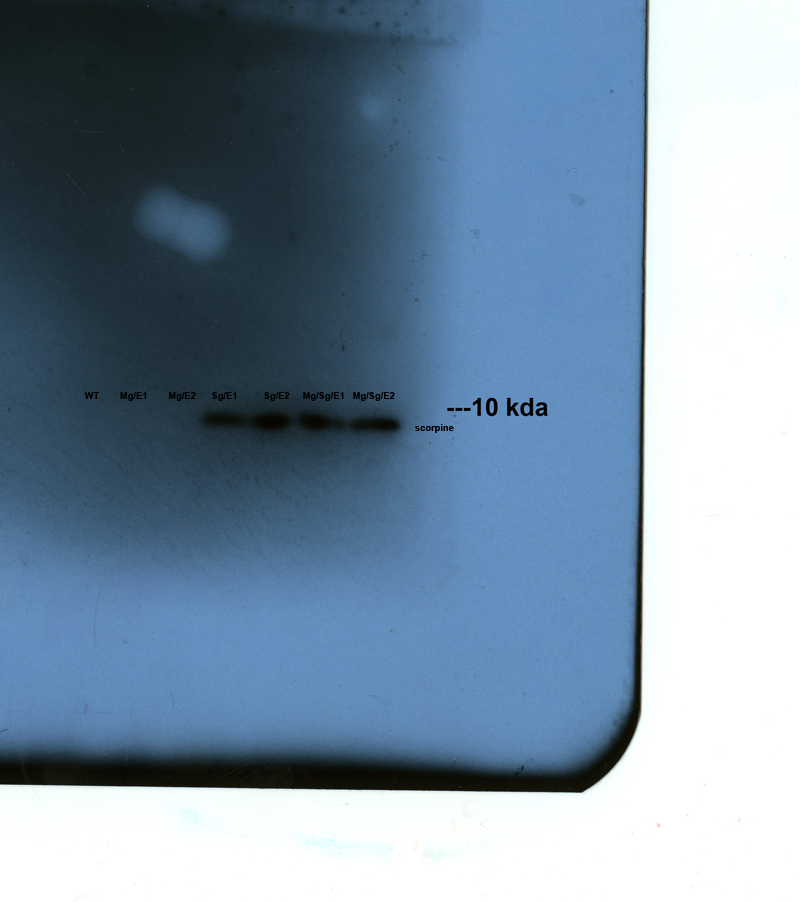

Supplement: Figure 1—source data 1. — Pictures of ‘Figure 1B-WT-blue field.tif,’ ‘Figure 1B-WT-red field.tif,’ and ‘Figure 1B-WT-yellow field.tif’ are original images of WT mosquito eye through blue, red, and yellow fluorescent filter, respectively; pictures of ‘Figure 1B-Mg-blue field.jpg,’ ‘Figure 1B-Mg-red field.jpg,’ and ‘Figure 1B-Mg-yellow field.jpg’ are original images of Mg mosquito line eye through blue, red, and yellow fluorescent filter, respectively; pictures of ‘Figure 1B-Sg-blue field.jpg,’ ‘Figure 1B-Sg-red field.jpg,’ and ‘-Figure 1B-Sg-yellow field.jpg’ are original images of Sg mosquito line eye through blue, red, and yellow fluorescent filter, respectively; pictures of ‘Figure 1B-E-blue field.jpg,’ ‘Figure 1B-E-red field.jpg,’ and ‘Figure 1B-E-yellow field.jpg’ are original images of E mosquito line eye through blue, red, and yellow fluorescent filter, respectively; pictures of Figure 1B-Mg-E-blue field.jpg, Figure 1B-Mg-E-red field.jpg, and Figure 1B-Mg-E-yellow field.jpg are original images of Mg/E mosquito line eye through blue, red, and yellow fluorescent filter, respectively; pictures of ‘Figure 1B-Sg-E-blue field.jpg,’ ‘Figure 1B-Sg-E-red field.jpg,’ and ‘Figure 1B-Sg-E-yellow field.jpg’ are original images of Sg/E mosquito line eye through blue, red, and yellow fluorescent filter, respectively; pictures of ‘Figure 1B-Mg+Sg-E-blue field.jpg,’ ‘Figure 1B-Mg+Sg-E-red field,’ and ‘Figure 1B-Mg+Sg-E-yellow field’ are original images of Mg/Sg/E mosquito line eye through blue, red, and yellow fluorescent filter, respectively. ‘Figure 1C and D-source data-RT-PCR data.xlsx’ is the original data for Figure 1C and D; ‘Figure 1C and D-gene expression.pzf’ shows Figure 1C and D were generated with GraphPad Prism. Pictures of ‘Figure 1E-western blot-MP2 in midgut.tif,’ ‘Figure 1E-western blot-Scorpine in midgut.tif,’ and ‘Figure 1E-western blot-α-tubulin in midgut.tif’ are original image of Western blots detected with mouse anti-MP2, mouse anti-scorpine, and rabbit anti-α-tubulin antibody. P [file elife-77584-fig1-data1.zip › Fig 1-source data/Fig 1F-western blot-Scorpine in salivary gland.tif]

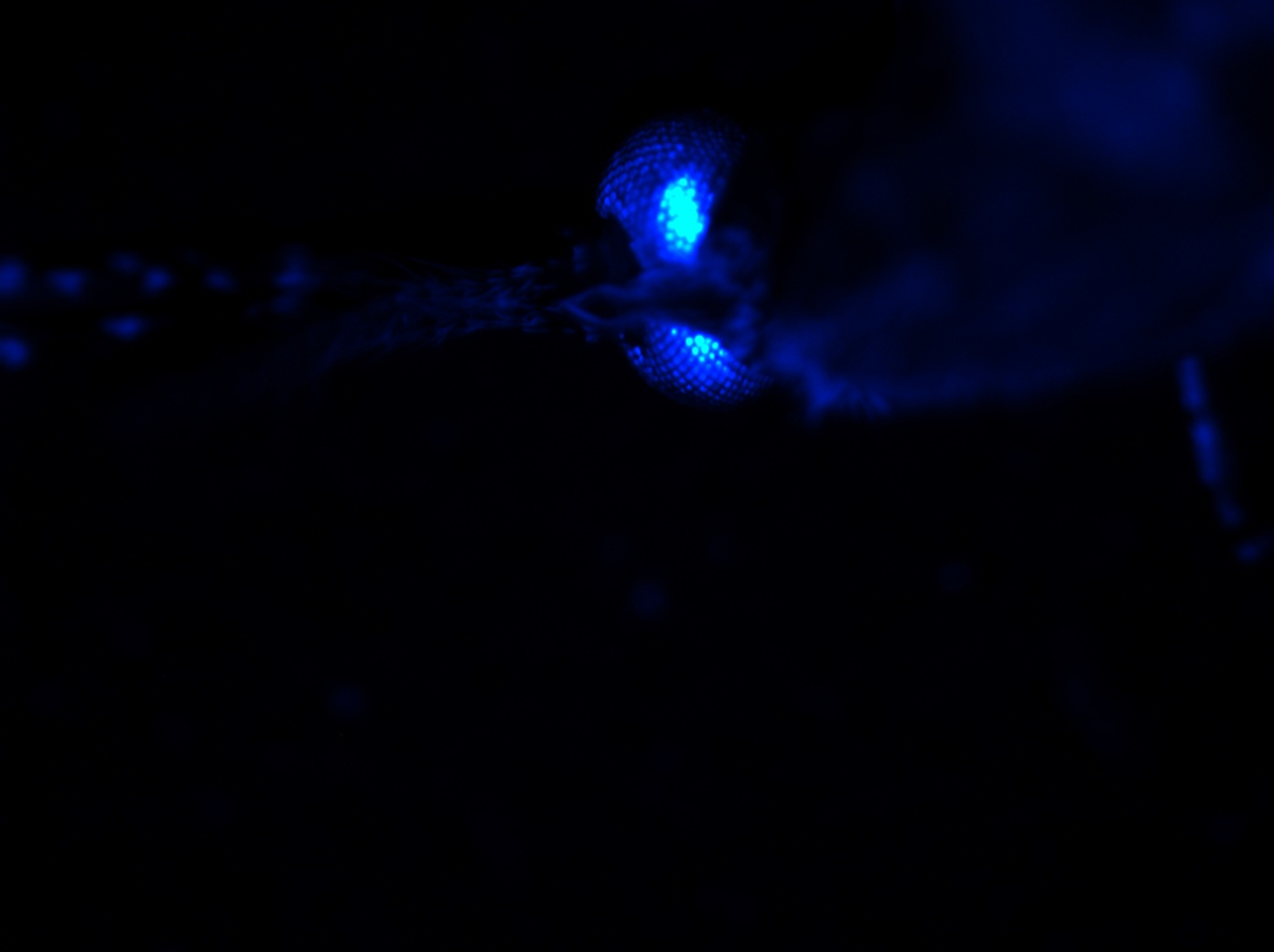

Supplement: Figure 1—source data 1. — Pictures of ‘Figure 1B-WT-blue field.tif,’ ‘Figure 1B-WT-red field.tif,’ and ‘Figure 1B-WT-yellow field.tif’ are original images of WT mosquito eye through blue, red, and yellow fluorescent filter, respectively; pictures of ‘Figure 1B-Mg-blue field.jpg,’ ‘Figure 1B-Mg-red field.jpg,’ and ‘Figure 1B-Mg-yellow field.jpg’ are original images of Mg mosquito line eye through blue, red, and yellow fluorescent filter, respectively; pictures of ‘Figure 1B-Sg-blue field.jpg,’ ‘Figure 1B-Sg-red field.jpg,’ and ‘-Figure 1B-Sg-yellow field.jpg’ are original images of Sg mosquito line eye through blue, red, and yellow fluorescent filter, respectively; pictures of ‘Figure 1B-E-blue field.jpg,’ ‘Figure 1B-E-red field.jpg,’ and ‘Figure 1B-E-yellow field.jpg’ are original images of E mosquito line eye through blue, red, and yellow fluorescent filter, respectively; pictures of Figure 1B-Mg-E-blue field.jpg, Figure 1B-Mg-E-red field.jpg, and Figure 1B-Mg-E-yellow field.jpg are original images of Mg/E mosquito line eye through blue, red, and yellow fluorescent filter, respectively; pictures of ‘Figure 1B-Sg-E-blue field.jpg,’ ‘Figure 1B-Sg-E-red field.jpg,’ and ‘Figure 1B-Sg-E-yellow field.jpg’ are original images of Sg/E mosquito line eye through blue, red, and yellow fluorescent filter, respectively; pictures of ‘Figure 1B-Mg+Sg-E-blue field.jpg,’ ‘Figure 1B-Mg+Sg-E-red field,’ and ‘Figure 1B-Mg+Sg-E-yellow field’ are original images of Mg/Sg/E mosquito line eye through blue, red, and yellow fluorescent filter, respectively. ‘Figure 1C and D-source data-RT-PCR data.xlsx’ is the original data for Figure 1C and D; ‘Figure 1C and D-gene expression.pzf’ shows Figure 1C and D were generated with GraphPad Prism. Pictures of ‘Figure 1E-western blot-MP2 in midgut.tif,’ ‘Figure 1E-western blot-Scorpine in midgut.tif,’ and ‘Figure 1E-western blot-α-tubulin in midgut.tif’ are original image of Western blots detected with mouse anti-MP2, mouse anti-scorpine, and rabbit anti-α-tubulin antibody. P [file elife-77584-fig1-data1.zip › Fig 1-source data/Fig1B-Mg+Sg-E line-blue field.jpg]

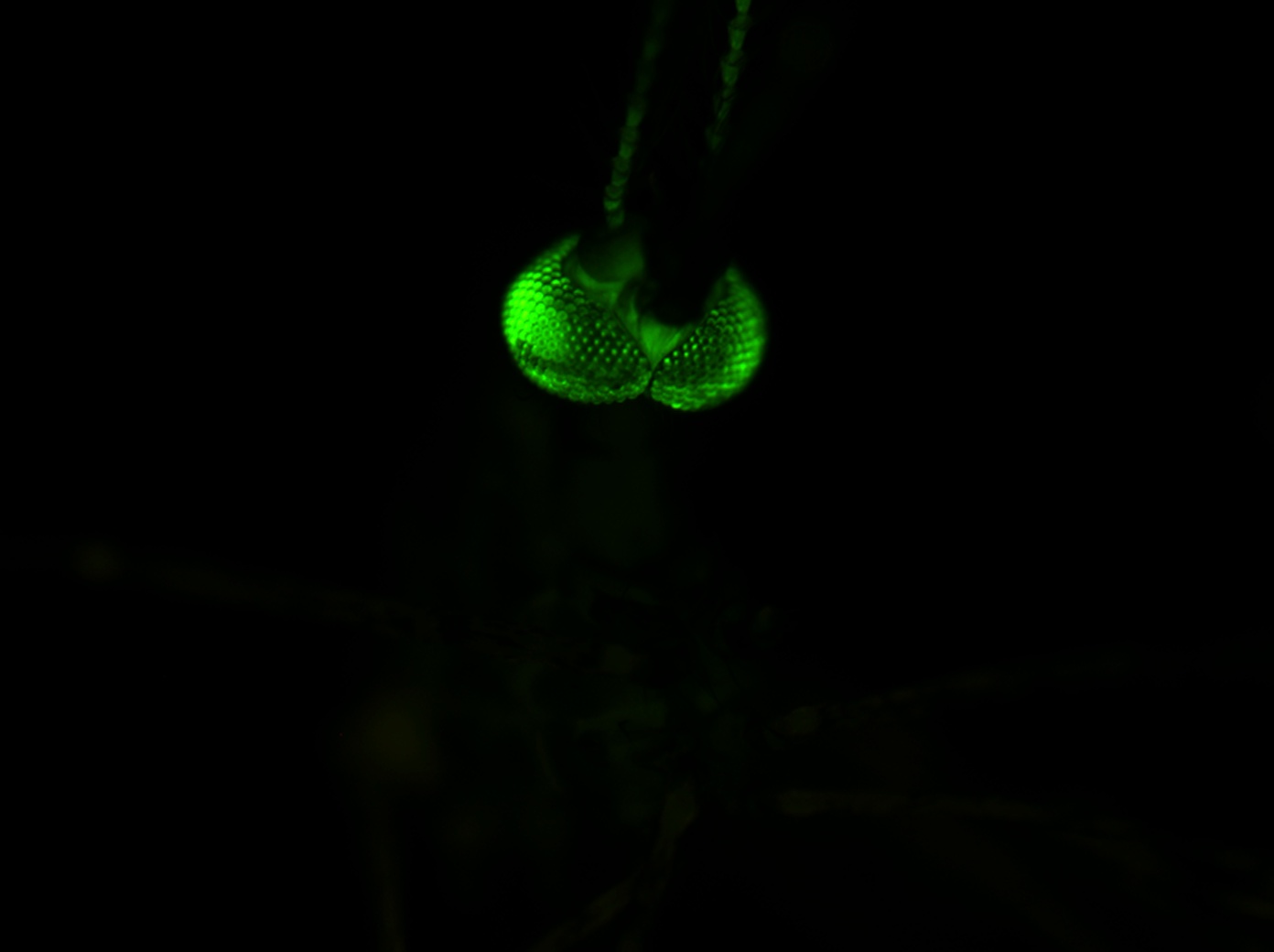

Supplement: Figure 1—source data 1. — Pictures of ‘Figure 1B-WT-blue field.tif,’ ‘Figure 1B-WT-red field.tif,’ and ‘Figure 1B-WT-yellow field.tif’ are original images of WT mosquito eye through blue, red, and yellow fluorescent filter, respectively; pictures of ‘Figure 1B-Mg-blue field.jpg,’ ‘Figure 1B-Mg-red field.jpg,’ and ‘Figure 1B-Mg-yellow field.jpg’ are original images of Mg mosquito line eye through blue, red, and yellow fluorescent filter, respectively; pictures of ‘Figure 1B-Sg-blue field.jpg,’ ‘Figure 1B-Sg-red field.jpg,’ and ‘-Figure 1B-Sg-yellow field.jpg’ are original images of Sg mosquito line eye through blue, red, and yellow fluorescent filter, respectively; pictures of ‘Figure 1B-E-blue field.jpg,’ ‘Figure 1B-E-red field.jpg,’ and ‘Figure 1B-E-yellow field.jpg’ are original images of E mosquito line eye through blue, red, and yellow fluorescent filter, respectively; pictures of Figure 1B-Mg-E-blue field.jpg, Figure 1B-Mg-E-red field.jpg, and Figure 1B-Mg-E-yellow field.jpg are original images of Mg/E mosquito line eye through blue, red, and yellow fluorescent filter, respectively; pictures of ‘Figure 1B-Sg-E-blue field.jpg,’ ‘Figure 1B-Sg-E-red field.jpg,’ and ‘Figure 1B-Sg-E-yellow field.jpg’ are original images of Sg/E mosquito line eye through blue, red, and yellow fluorescent filter, respectively; pictures of ‘Figure 1B-Mg+Sg-E-blue field.jpg,’ ‘Figure 1B-Mg+Sg-E-red field,’ and ‘Figure 1B-Mg+Sg-E-yellow field’ are original images of Mg/Sg/E mosquito line eye through blue, red, and yellow fluorescent filter, respectively. ‘Figure 1C and D-source data-RT-PCR data.xlsx’ is the original data for Figure 1C and D; ‘Figure 1C and D-gene expression.pzf’ shows Figure 1C and D were generated with GraphPad Prism. Pictures of ‘Figure 1E-western blot-MP2 in midgut.tif,’ ‘Figure 1E-western blot-Scorpine in midgut.tif,’ and ‘Figure 1E-western blot-α-tubulin in midgut.tif’ are original image of Western blots detected with mouse anti-MP2, mouse anti-scorpine, and rabbit anti-α-tubulin antibody. P [file elife-77584-fig1-data1.zip › Fig 1-source data/Fig1B-Sg-E line-yellow field.jpg]

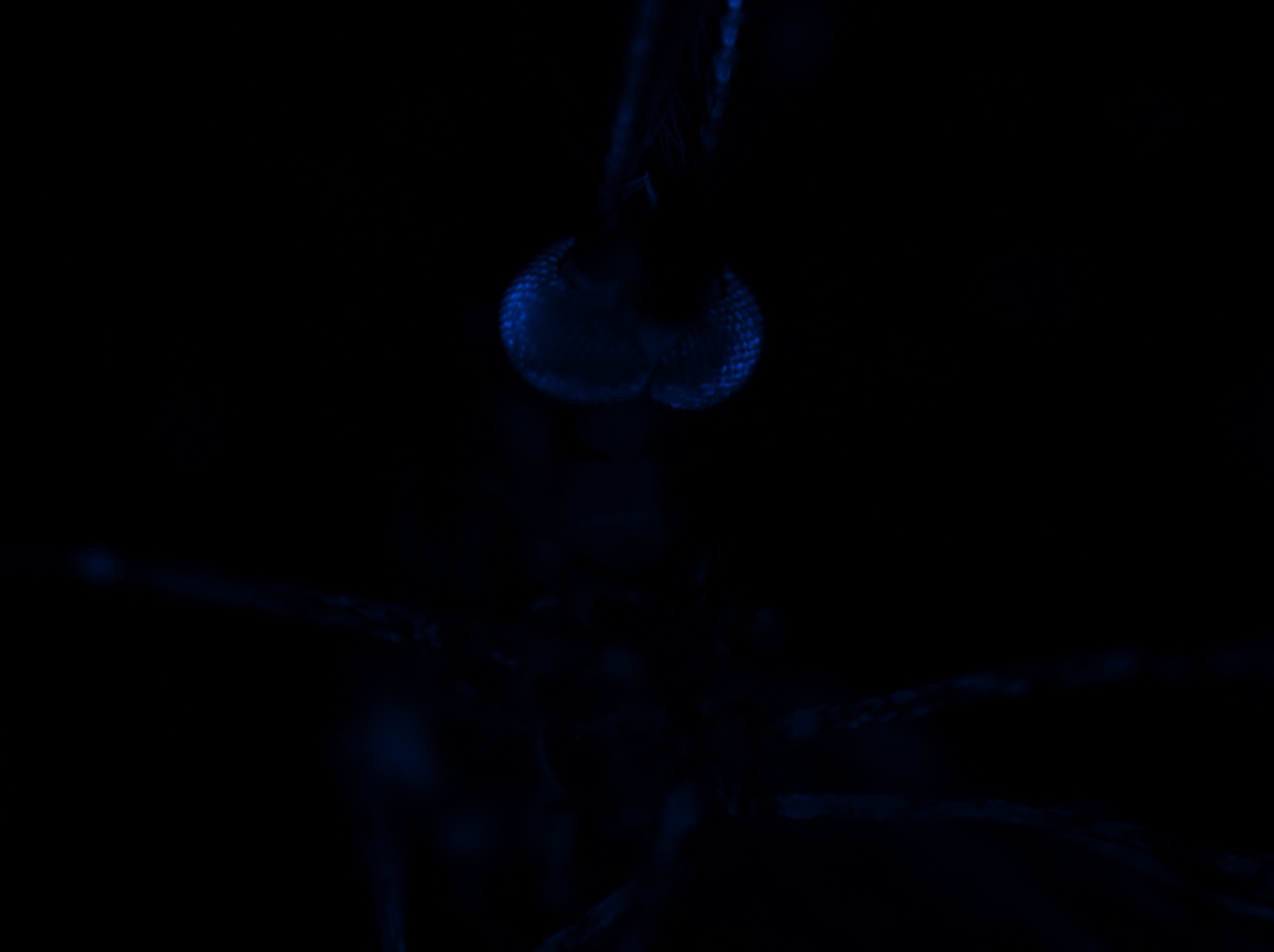

Supplement: Figure 1—source data 1. — Pictures of ‘Figure 1B-WT-blue field.tif,’ ‘Figure 1B-WT-red field.tif,’ and ‘Figure 1B-WT-yellow field.tif’ are original images of WT mosquito eye through blue, red, and yellow fluorescent filter, respectively; pictures of ‘Figure 1B-Mg-blue field.jpg,’ ‘Figure 1B-Mg-red field.jpg,’ and ‘Figure 1B-Mg-yellow field.jpg’ are original images of Mg mosquito line eye through blue, red, and yellow fluorescent filter, respectively; pictures of ‘Figure 1B-Sg-blue field.jpg,’ ‘Figure 1B-Sg-red field.jpg,’ and ‘-Figure 1B-Sg-yellow field.jpg’ are original images of Sg mosquito line eye through blue, red, and yellow fluorescent filter, respectively; pictures of ‘Figure 1B-E-blue field.jpg,’ ‘Figure 1B-E-red field.jpg,’ and ‘Figure 1B-E-yellow field.jpg’ are original images of E mosquito line eye through blue, red, and yellow fluorescent filter, respectively; pictures of Figure 1B-Mg-E-blue field.jpg, Figure 1B-Mg-E-red field.jpg, and Figure 1B-Mg-E-yellow field.jpg are original images of Mg/E mosquito line eye through blue, red, and yellow fluorescent filter, respectively; pictures of ‘Figure 1B-Sg-E-blue field.jpg,’ ‘Figure 1B-Sg-E-red field.jpg,’ and ‘Figure 1B-Sg-E-yellow field.jpg’ are original images of Sg/E mosquito line eye through blue, red, and yellow fluorescent filter, respectively; pictures of ‘Figure 1B-Mg+Sg-E-blue field.jpg,’ ‘Figure 1B-Mg+Sg-E-red field,’ and ‘Figure 1B-Mg+Sg-E-yellow field’ are original images of Mg/Sg/E mosquito line eye through blue, red, and yellow fluorescent filter, respectively. ‘Figure 1C and D-source data-RT-PCR data.xlsx’ is the original data for Figure 1C and D; ‘Figure 1C and D-gene expression.pzf’ shows Figure 1C and D were generated with GraphPad Prism. Pictures of ‘Figure 1E-western blot-MP2 in midgut.tif,’ ‘Figure 1E-western blot-Scorpine in midgut.tif,’ and ‘Figure 1E-western blot-α-tubulin in midgut.tif’ are original image of Western blots detected with mouse anti-MP2, mouse anti-scorpine, and rabbit anti-α-tubulin antibody. P [file elife-77584-fig1-data1.zip › Fig 1-source data/Fig1B-Sg-E line-blue field.jpg]

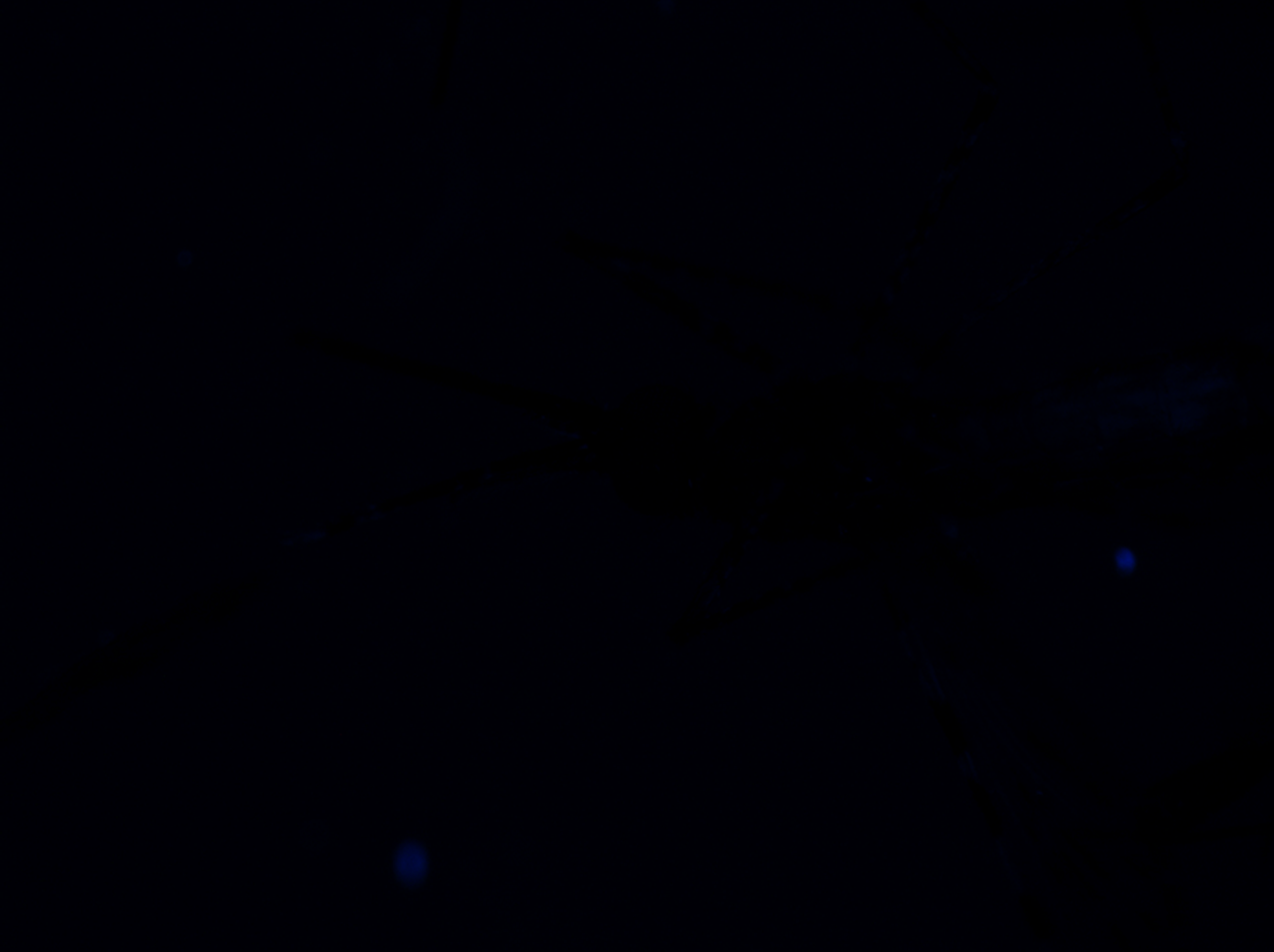

Supplement: Figure 1—source data 1. — Pictures of ‘Figure 1B-WT-blue field.tif,’ ‘Figure 1B-WT-red field.tif,’ and ‘Figure 1B-WT-yellow field.tif’ are original images of WT mosquito eye through blue, red, and yellow fluorescent filter, respectively; pictures of ‘Figure 1B-Mg-blue field.jpg,’ ‘Figure 1B-Mg-red field.jpg,’ and ‘Figure 1B-Mg-yellow field.jpg’ are original images of Mg mosquito line eye through blue, red, and yellow fluorescent filter, respectively; pictures of ‘Figure 1B-Sg-blue field.jpg,’ ‘Figure 1B-Sg-red field.jpg,’ and ‘-Figure 1B-Sg-yellow field.jpg’ are original images of Sg mosquito line eye through blue, red, and yellow fluorescent filter, respectively; pictures of ‘Figure 1B-E-blue field.jpg,’ ‘Figure 1B-E-red field.jpg,’ and ‘Figure 1B-E-yellow field.jpg’ are original images of E mosquito line eye through blue, red, and yellow fluorescent filter, respectively; pictures of Figure 1B-Mg-E-blue field.jpg, Figure 1B-Mg-E-red field.jpg, and Figure 1B-Mg-E-yellow field.jpg are original images of Mg/E mosquito line eye through blue, red, and yellow fluorescent filter, respectively; pictures of ‘Figure 1B-Sg-E-blue field.jpg,’ ‘Figure 1B-Sg-E-red field.jpg,’ and ‘Figure 1B-Sg-E-yellow field.jpg’ are original images of Sg/E mosquito line eye through blue, red, and yellow fluorescent filter, respectively; pictures of ‘Figure 1B-Mg+Sg-E-blue field.jpg,’ ‘Figure 1B-Mg+Sg-E-red field,’ and ‘Figure 1B-Mg+Sg-E-yellow field’ are original images of Mg/Sg/E mosquito line eye through blue, red, and yellow fluorescent filter, respectively. ‘Figure 1C and D-source data-RT-PCR data.xlsx’ is the original data for Figure 1C and D; ‘Figure 1C and D-gene expression.pzf’ shows Figure 1C and D were generated with GraphPad Prism. Pictures of ‘Figure 1E-western blot-MP2 in midgut.tif,’ ‘Figure 1E-western blot-Scorpine in midgut.tif,’ and ‘Figure 1E-western blot-α-tubulin in midgut.tif’ are original image of Western blots detected with mouse anti-MP2, mouse anti-scorpine, and rabbit anti-α-tubulin antibody. P [file elife-77584-fig1-data1.zip › Fig 1-source data/Fig1B-Sg line-blue field.tif]

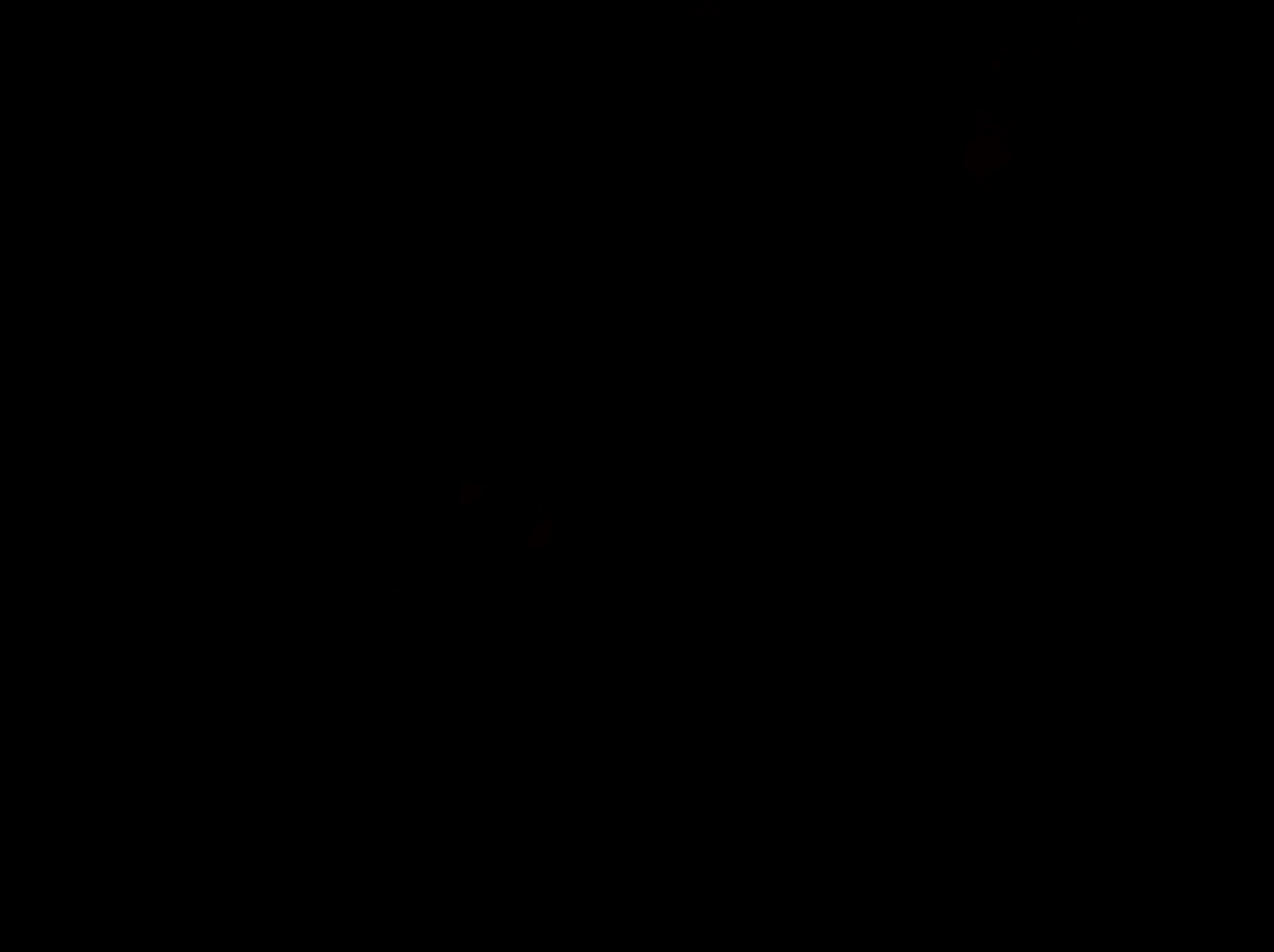

Supplement: Figure 1—source data 1. — Pictures of ‘Figure 1B-WT-blue field.tif,’ ‘Figure 1B-WT-red field.tif,’ and ‘Figure 1B-WT-yellow field.tif’ are original images of WT mosquito eye through blue, red, and yellow fluorescent filter, respectively; pictures of ‘Figure 1B-Mg-blue field.jpg,’ ‘Figure 1B-Mg-red field.jpg,’ and ‘Figure 1B-Mg-yellow field.jpg’ are original images of Mg mosquito line eye through blue, red, and yellow fluorescent filter, respectively; pictures of ‘Figure 1B-Sg-blue field.jpg,’ ‘Figure 1B-Sg-red field.jpg,’ and ‘-Figure 1B-Sg-yellow field.jpg’ are original images of Sg mosquito line eye through blue, red, and yellow fluorescent filter, respectively; pictures of ‘Figure 1B-E-blue field.jpg,’ ‘Figure 1B-E-red field.jpg,’ and ‘Figure 1B-E-yellow field.jpg’ are original images of E mosquito line eye through blue, red, and yellow fluorescent filter, respectively; pictures of Figure 1B-Mg-E-blue field.jpg, Figure 1B-Mg-E-red field.jpg, and Figure 1B-Mg-E-yellow field.jpg are original images of Mg/E mosquito line eye through blue, red, and yellow fluorescent filter, respectively; pictures of ‘Figure 1B-Sg-E-blue field.jpg,’ ‘Figure 1B-Sg-E-red field.jpg,’ and ‘Figure 1B-Sg-E-yellow field.jpg’ are original images of Sg/E mosquito line eye through blue, red, and yellow fluorescent filter, respectively; pictures of ‘Figure 1B-Mg+Sg-E-blue field.jpg,’ ‘Figure 1B-Mg+Sg-E-red field,’ and ‘Figure 1B-Mg+Sg-E-yellow field’ are original images of Mg/Sg/E mosquito line eye through blue, red, and yellow fluorescent filter, respectively. ‘Figure 1C and D-source data-RT-PCR data.xlsx’ is the original data for Figure 1C and D; ‘Figure 1C and D-gene expression.pzf’ shows Figure 1C and D were generated with GraphPad Prism. Pictures of ‘Figure 1E-western blot-MP2 in midgut.tif,’ ‘Figure 1E-western blot-Scorpine in midgut.tif,’ and ‘Figure 1E-western blot-α-tubulin in midgut.tif’ are original image of Western blots detected with mouse anti-MP2, mouse anti-scorpine, and rabbit anti-α-tubulin antibody. P [file elife-77584-fig1-data1.zip › Fig 1-source data/Fig1B-Sg-E line-red field.jpg]

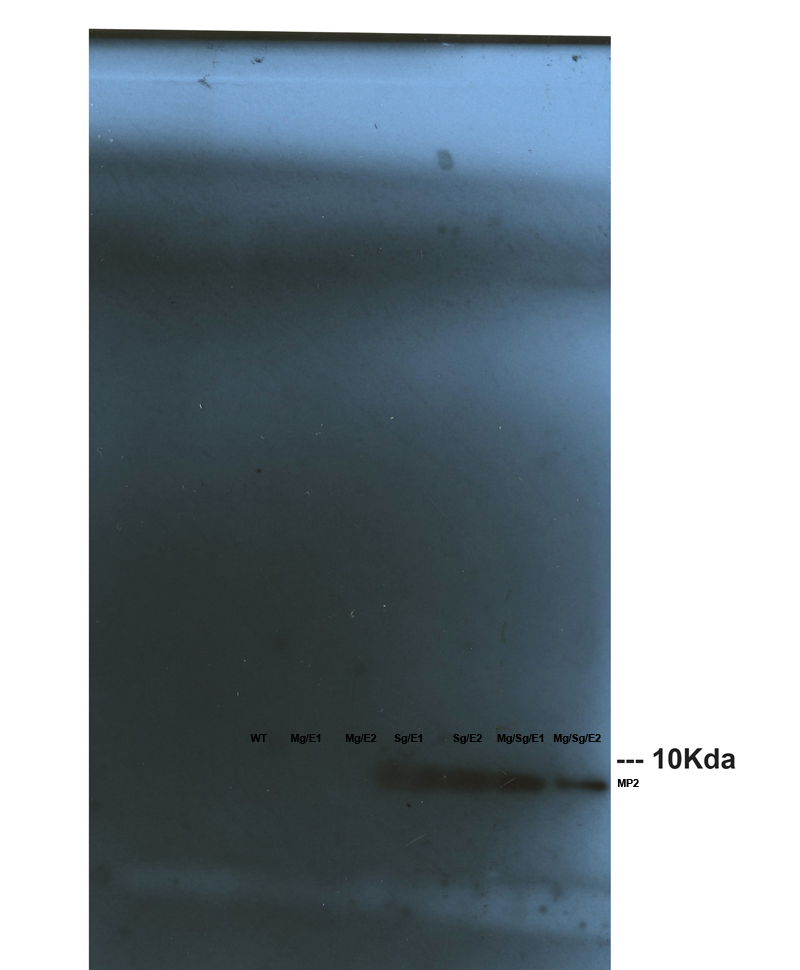

Supplement: Figure 1—source data 1. — Pictures of ‘Figure 1B-WT-blue field.tif,’ ‘Figure 1B-WT-red field.tif,’ and ‘Figure 1B-WT-yellow field.tif’ are original images of WT mosquito eye through blue, red, and yellow fluorescent filter, respectively; pictures of ‘Figure 1B-Mg-blue field.jpg,’ ‘Figure 1B-Mg-red field.jpg,’ and ‘Figure 1B-Mg-yellow field.jpg’ are original images of Mg mosquito line eye through blue, red, and yellow fluorescent filter, respectively; pictures of ‘Figure 1B-Sg-blue field.jpg,’ ‘Figure 1B-Sg-red field.jpg,’ and ‘-Figure 1B-Sg-yellow field.jpg’ are original images of Sg mosquito line eye through blue, red, and yellow fluorescent filter, respectively; pictures of ‘Figure 1B-E-blue field.jpg,’ ‘Figure 1B-E-red field.jpg,’ and ‘Figure 1B-E-yellow field.jpg’ are original images of E mosquito line eye through blue, red, and yellow fluorescent filter, respectively; pictures of Figure 1B-Mg-E-blue field.jpg, Figure 1B-Mg-E-red field.jpg, and Figure 1B-Mg-E-yellow field.jpg are original images of Mg/E mosquito line eye through blue, red, and yellow fluorescent filter, respectively; pictures of ‘Figure 1B-Sg-E-blue field.jpg,’ ‘Figure 1B-Sg-E-red field.jpg,’ and ‘Figure 1B-Sg-E-yellow field.jpg’ are original images of Sg/E mosquito line eye through blue, red, and yellow fluorescent filter, respectively; pictures of ‘Figure 1B-Mg+Sg-E-blue field.jpg,’ ‘Figure 1B-Mg+Sg-E-red field,’ and ‘Figure 1B-Mg+Sg-E-yellow field’ are original images of Mg/Sg/E mosquito line eye through blue, red, and yellow fluorescent filter, respectively. ‘Figure 1C and D-source data-RT-PCR data.xlsx’ is the original data for Figure 1C and D; ‘Figure 1C and D-gene expression.pzf’ shows Figure 1C and D were generated with GraphPad Prism. Pictures of ‘Figure 1E-western blot-MP2 in midgut.tif,’ ‘Figure 1E-western blot-Scorpine in midgut.tif,’ and ‘Figure 1E-western blot-α-tubulin in midgut.tif’ are original image of Western blots detected with mouse anti-MP2, mouse anti-scorpine, and rabbit anti-α-tubulin antibody. P [file elife-77584-fig1-data1.zip › Fig 1-source data/Fig 1F-western blot-MP2 in salivary gland.tif]

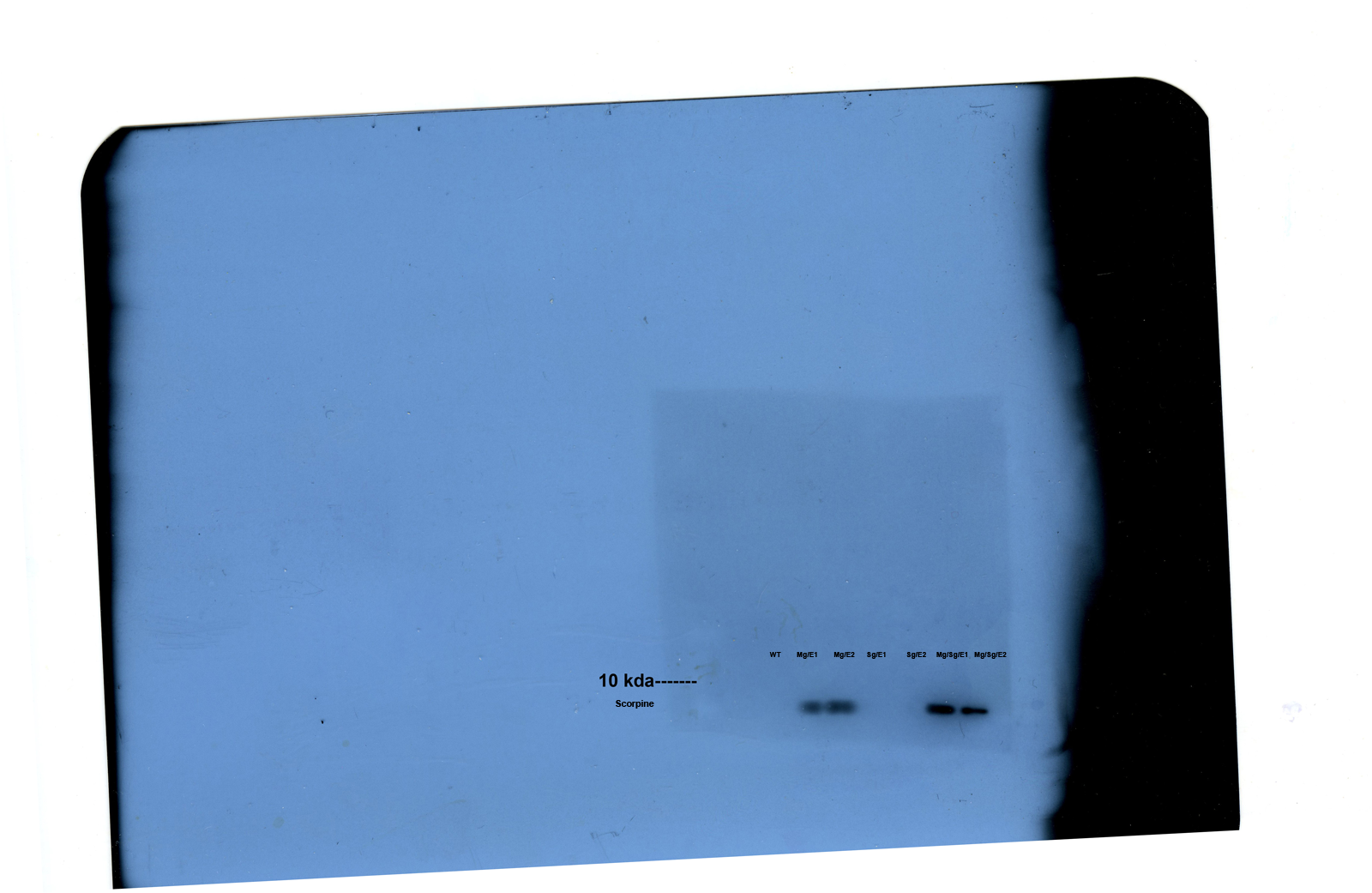

Supplement: Figure 1—source data 1. — Pictures of ‘Figure 1B-WT-blue field.tif,’ ‘Figure 1B-WT-red field.tif,’ and ‘Figure 1B-WT-yellow field.tif’ are original images of WT mosquito eye through blue, red, and yellow fluorescent filter, respectively; pictures of ‘Figure 1B-Mg-blue field.jpg,’ ‘Figure 1B-Mg-red field.jpg,’ and ‘Figure 1B-Mg-yellow field.jpg’ are original images of Mg mosquito line eye through blue, red, and yellow fluorescent filter, respectively; pictures of ‘Figure 1B-Sg-blue field.jpg,’ ‘Figure 1B-Sg-red field.jpg,’ and ‘-Figure 1B-Sg-yellow field.jpg’ are original images of Sg mosquito line eye through blue, red, and yellow fluorescent filter, respectively; pictures of ‘Figure 1B-E-blue field.jpg,’ ‘Figure 1B-E-red field.jpg,’ and ‘Figure 1B-E-yellow field.jpg’ are original images of E mosquito line eye through blue, red, and yellow fluorescent filter, respectively; pictures of Figure 1B-Mg-E-blue field.jpg, Figure 1B-Mg-E-red field.jpg, and Figure 1B-Mg-E-yellow field.jpg are original images of Mg/E mosquito line eye through blue, red, and yellow fluorescent filter, respectively; pictures of ‘Figure 1B-Sg-E-blue field.jpg,’ ‘Figure 1B-Sg-E-red field.jpg,’ and ‘Figure 1B-Sg-E-yellow field.jpg’ are original images of Sg/E mosquito line eye through blue, red, and yellow fluorescent filter, respectively; pictures of ‘Figure 1B-Mg+Sg-E-blue field.jpg,’ ‘Figure 1B-Mg+Sg-E-red field,’ and ‘Figure 1B-Mg+Sg-E-yellow field’ are original images of Mg/Sg/E mosquito line eye through blue, red, and yellow fluorescent filter, respectively. ‘Figure 1C and D-source data-RT-PCR data.xlsx’ is the original data for Figure 1C and D; ‘Figure 1C and D-gene expression.pzf’ shows Figure 1C and D were generated with GraphPad Prism. Pictures of ‘Figure 1E-western blot-MP2 in midgut.tif,’ ‘Figure 1E-western blot-Scorpine in midgut.tif,’ and ‘Figure 1E-western blot-α-tubulin in midgut.tif’ are original image of Western blots detected with mouse anti-MP2, mouse anti-scorpine, and rabbit anti-α-tubulin antibody. P [file elife-77584-fig1-data1.zip › Fig 1-source data/Fig1E-western blot-Scorpine in midgut.tif]

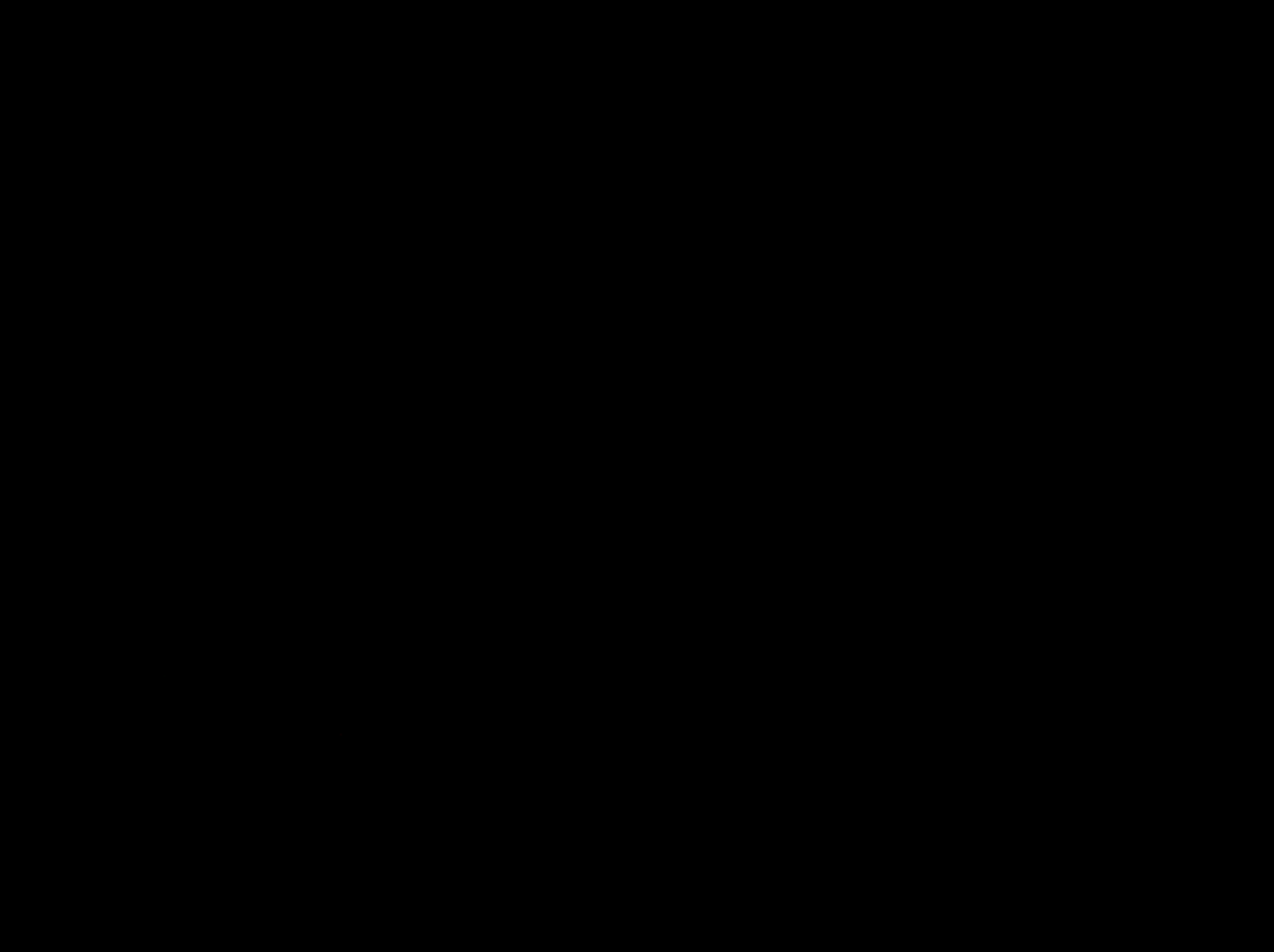

Supplement: Figure 1—source data 1. — Pictures of ‘Figure 1B-WT-blue field.tif,’ ‘Figure 1B-WT-red field.tif,’ and ‘Figure 1B-WT-yellow field.tif’ are original images of WT mosquito eye through blue, red, and yellow fluorescent filter, respectively; pictures of ‘Figure 1B-Mg-blue field.jpg,’ ‘Figure 1B-Mg-red field.jpg,’ and ‘Figure 1B-Mg-yellow field.jpg’ are original images of Mg mosquito line eye through blue, red, and yellow fluorescent filter, respectively; pictures of ‘Figure 1B-Sg-blue field.jpg,’ ‘Figure 1B-Sg-red field.jpg,’ and ‘-Figure 1B-Sg-yellow field.jpg’ are original images of Sg mosquito line eye through blue, red, and yellow fluorescent filter, respectively; pictures of ‘Figure 1B-E-blue field.jpg,’ ‘Figure 1B-E-red field.jpg,’ and ‘Figure 1B-E-yellow field.jpg’ are original images of E mosquito line eye through blue, red, and yellow fluorescent filter, respectively; pictures of Figure 1B-Mg-E-blue field.jpg, Figure 1B-Mg-E-red field.jpg, and Figure 1B-Mg-E-yellow field.jpg are original images of Mg/E mosquito line eye through blue, red, and yellow fluorescent filter, respectively; pictures of ‘Figure 1B-Sg-E-blue field.jpg,’ ‘Figure 1B-Sg-E-red field.jpg,’ and ‘Figure 1B-Sg-E-yellow field.jpg’ are original images of Sg/E mosquito line eye through blue, red, and yellow fluorescent filter, respectively; pictures of ‘Figure 1B-Mg+Sg-E-blue field.jpg,’ ‘Figure 1B-Mg+Sg-E-red field,’ and ‘Figure 1B-Mg+Sg-E-yellow field’ are original images of Mg/Sg/E mosquito line eye through blue, red, and yellow fluorescent filter, respectively. ‘Figure 1C and D-source data-RT-PCR data.xlsx’ is the original data for Figure 1C and D; ‘Figure 1C and D-gene expression.pzf’ shows Figure 1C and D were generated with GraphPad Prism. Pictures of ‘Figure 1E-western blot-MP2 in midgut.tif,’ ‘Figure 1E-western blot-Scorpine in midgut.tif,’ and ‘Figure 1E-western blot-α-tubulin in midgut.tif’ are original image of Western blots detected with mouse anti-MP2, mouse anti-scorpine, and rabbit anti-α-tubulin antibody. P [file elife-77584-fig1-data1.zip › Fig 1-source data/Fig1B-WT-yellow field.tif]

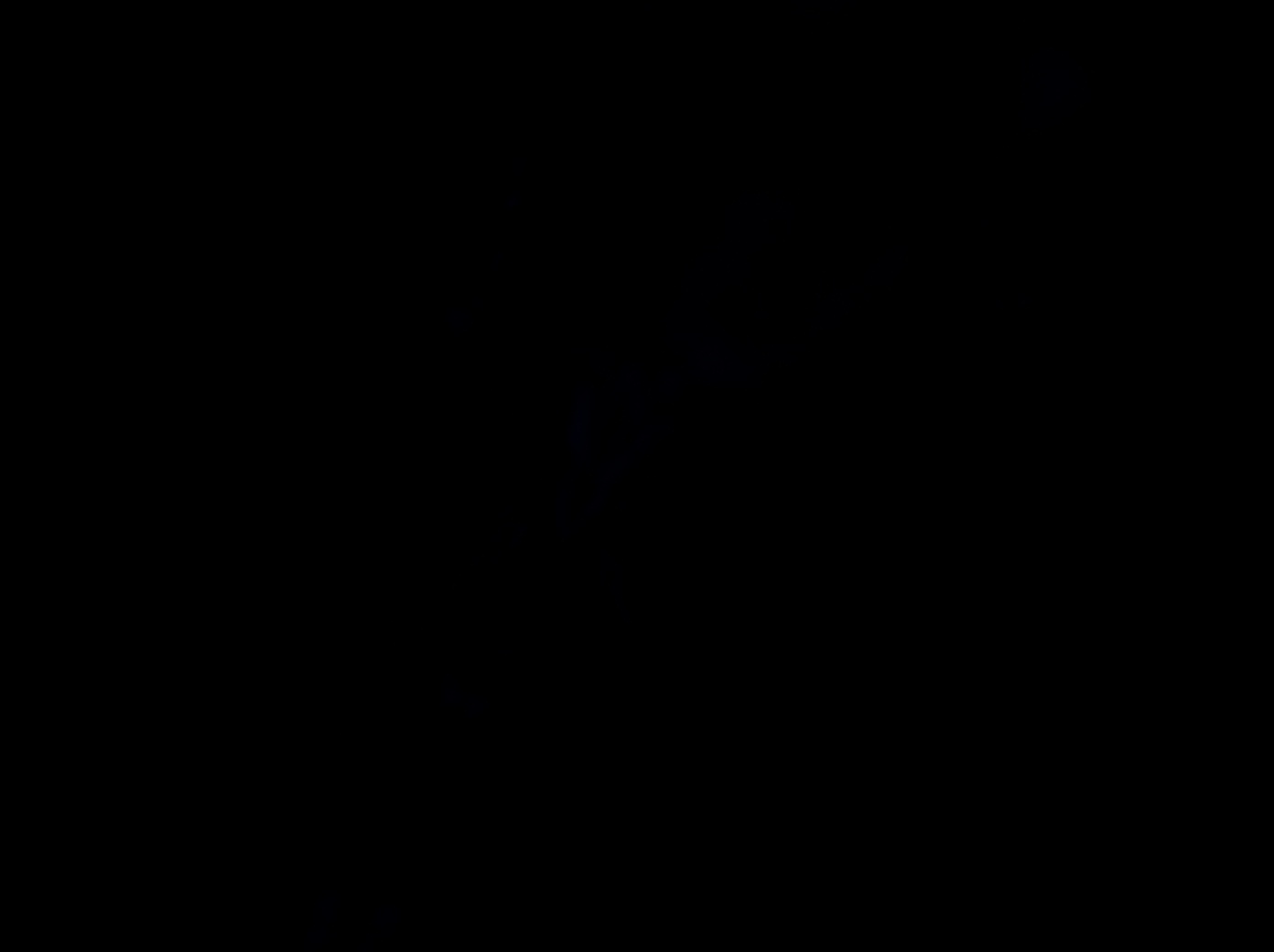

Supplement: Figure 1—source data 1. — Pictures of ‘Figure 1B-WT-blue field.tif,’ ‘Figure 1B-WT-red field.tif,’ and ‘Figure 1B-WT-yellow field.tif’ are original images of WT mosquito eye through blue, red, and yellow fluorescent filter, respectively; pictures of ‘Figure 1B-Mg-blue field.jpg,’ ‘Figure 1B-Mg-red field.jpg,’ and ‘Figure 1B-Mg-yellow field.jpg’ are original images of Mg mosquito line eye through blue, red, and yellow fluorescent filter, respectively; pictures of ‘Figure 1B-Sg-blue field.jpg,’ ‘Figure 1B-Sg-red field.jpg,’ and ‘-Figure 1B-Sg-yellow field.jpg’ are original images of Sg mosquito line eye through blue, red, and yellow fluorescent filter, respectively; pictures of ‘Figure 1B-E-blue field.jpg,’ ‘Figure 1B-E-red field.jpg,’ and ‘Figure 1B-E-yellow field.jpg’ are original images of E mosquito line eye through blue, red, and yellow fluorescent filter, respectively; pictures of Figure 1B-Mg-E-blue field.jpg, Figure 1B-Mg-E-red field.jpg, and Figure 1B-Mg-E-yellow field.jpg are original images of Mg/E mosquito line eye through blue, red, and yellow fluorescent filter, respectively; pictures of ‘Figure 1B-Sg-E-blue field.jpg,’ ‘Figure 1B-Sg-E-red field.jpg,’ and ‘Figure 1B-Sg-E-yellow field.jpg’ are original images of Sg/E mosquito line eye through blue, red, and yellow fluorescent filter, respectively; pictures of ‘Figure 1B-Mg+Sg-E-blue field.jpg,’ ‘Figure 1B-Mg+Sg-E-red field,’ and ‘Figure 1B-Mg+Sg-E-yellow field’ are original images of Mg/Sg/E mosquito line eye through blue, red, and yellow fluorescent filter, respectively. ‘Figure 1C and D-source data-RT-PCR data.xlsx’ is the original data for Figure 1C and D; ‘Figure 1C and D-gene expression.pzf’ shows Figure 1C and D were generated with GraphPad Prism. Pictures of ‘Figure 1E-western blot-MP2 in midgut.tif,’ ‘Figure 1E-western blot-Scorpine in midgut.tif,’ and ‘Figure 1E-western blot-α-tubulin in midgut.tif’ are original image of Western blots detected with mouse anti-MP2, mouse anti-scorpine, and rabbit anti-α-tubulin antibody. P [file elife-77584-fig1-data1.zip › Fig 1-source data/Fig1B-Mg line-blue field.jpg]

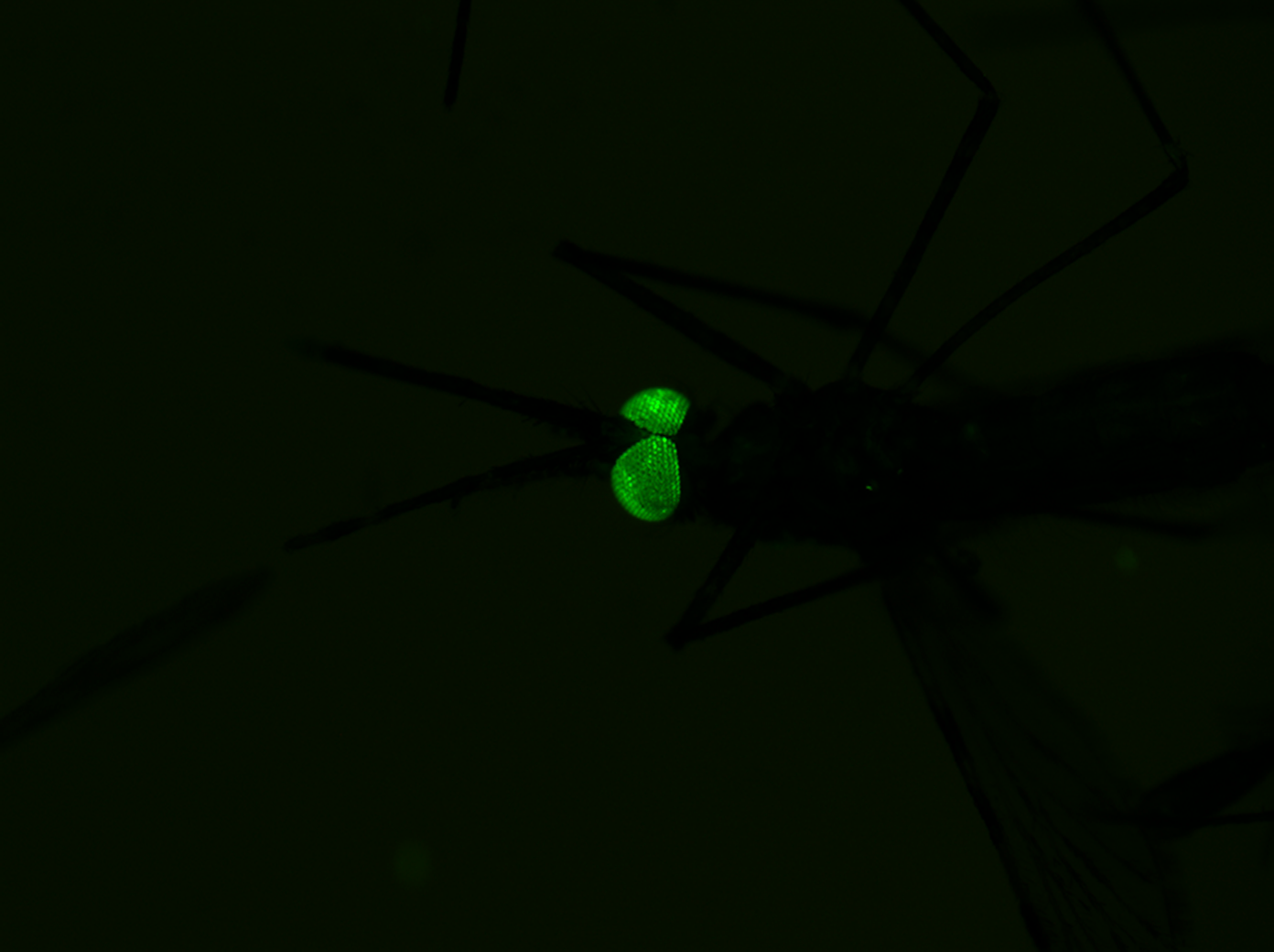

Supplement: Figure 1—source data 1. — Pictures of ‘Figure 1B-WT-blue field.tif,’ ‘Figure 1B-WT-red field.tif,’ and ‘Figure 1B-WT-yellow field.tif’ are original images of WT mosquito eye through blue, red, and yellow fluorescent filter, respectively; pictures of ‘Figure 1B-Mg-blue field.jpg,’ ‘Figure 1B-Mg-red field.jpg,’ and ‘Figure 1B-Mg-yellow field.jpg’ are original images of Mg mosquito line eye through blue, red, and yellow fluorescent filter, respectively; pictures of ‘Figure 1B-Sg-blue field.jpg,’ ‘Figure 1B-Sg-red field.jpg,’ and ‘-Figure 1B-Sg-yellow field.jpg’ are original images of Sg mosquito line eye through blue, red, and yellow fluorescent filter, respectively; pictures of ‘Figure 1B-E-blue field.jpg,’ ‘Figure 1B-E-red field.jpg,’ and ‘Figure 1B-E-yellow field.jpg’ are original images of E mosquito line eye through blue, red, and yellow fluorescent filter, respectively; pictures of Figure 1B-Mg-E-blue field.jpg, Figure 1B-Mg-E-red field.jpg, and Figure 1B-Mg-E-yellow field.jpg are original images of Mg/E mosquito line eye through blue, red, and yellow fluorescent filter, respectively; pictures of ‘Figure 1B-Sg-E-blue field.jpg,’ ‘Figure 1B-Sg-E-red field.jpg,’ and ‘Figure 1B-Sg-E-yellow field.jpg’ are original images of Sg/E mosquito line eye through blue, red, and yellow fluorescent filter, respectively; pictures of ‘Figure 1B-Mg+Sg-E-blue field.jpg,’ ‘Figure 1B-Mg+Sg-E-red field,’ and ‘Figure 1B-Mg+Sg-E-yellow field’ are original images of Mg/Sg/E mosquito line eye through blue, red, and yellow fluorescent filter, respectively. ‘Figure 1C and D-source data-RT-PCR data.xlsx’ is the original data for Figure 1C and D; ‘Figure 1C and D-gene expression.pzf’ shows Figure 1C and D were generated with GraphPad Prism. Pictures of ‘Figure 1E-western blot-MP2 in midgut.tif,’ ‘Figure 1E-western blot-Scorpine in midgut.tif,’ and ‘Figure 1E-western blot-α-tubulin in midgut.tif’ are original image of Western blots detected with mouse anti-MP2, mouse anti-scorpine, and rabbit anti-α-tubulin antibody. P [file elife-77584-fig1-data1.zip › Fig 1-source data/Fig1B-Sg line-yellow field.tif]

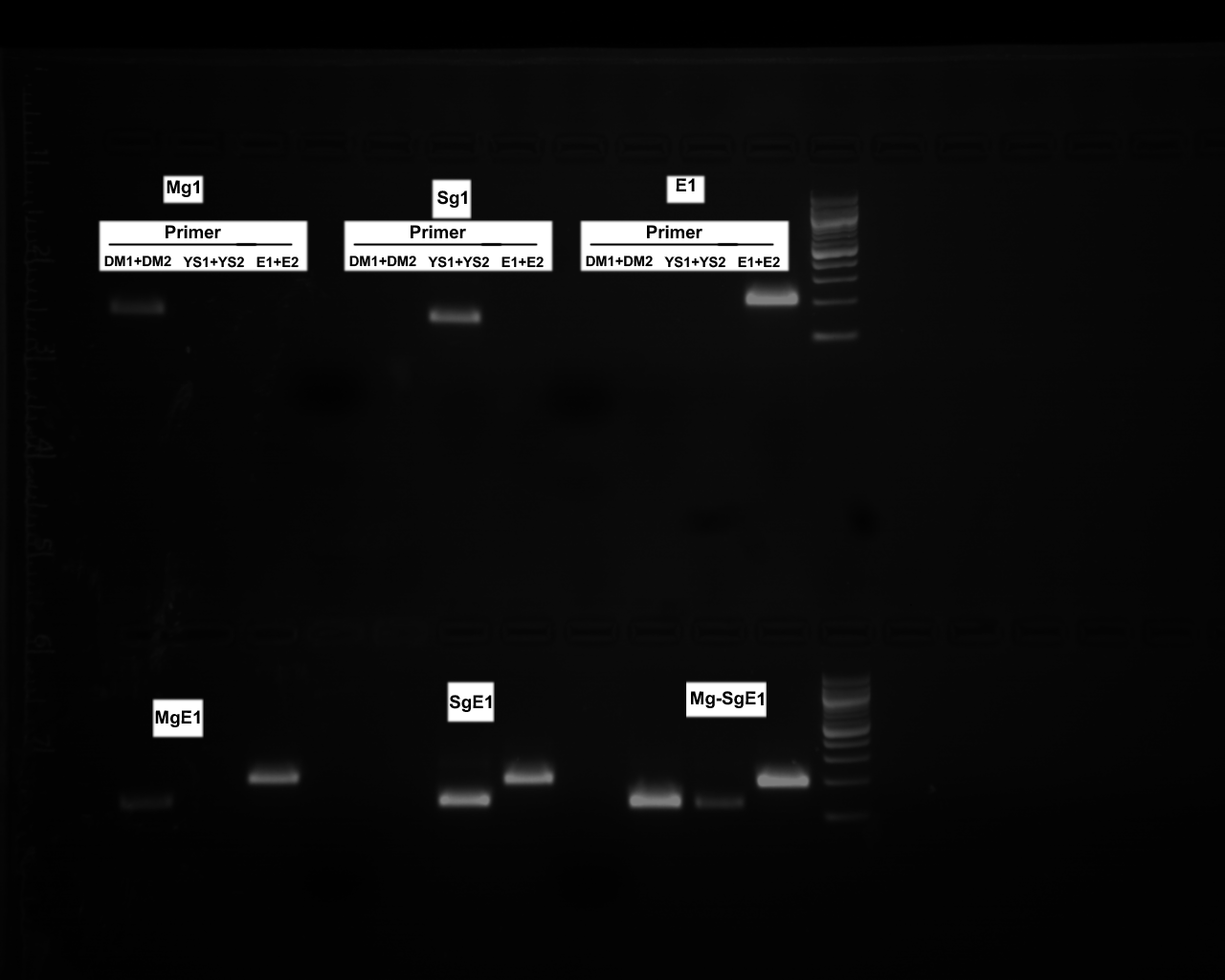

Supplement: Source data 1. [file elife-77584-data1.zip › Source data 28sep/Appendix 1-Figure 1-source data 28sep/Appendix 1-Figure 1-DNA gel of PCR verification 1.tif]

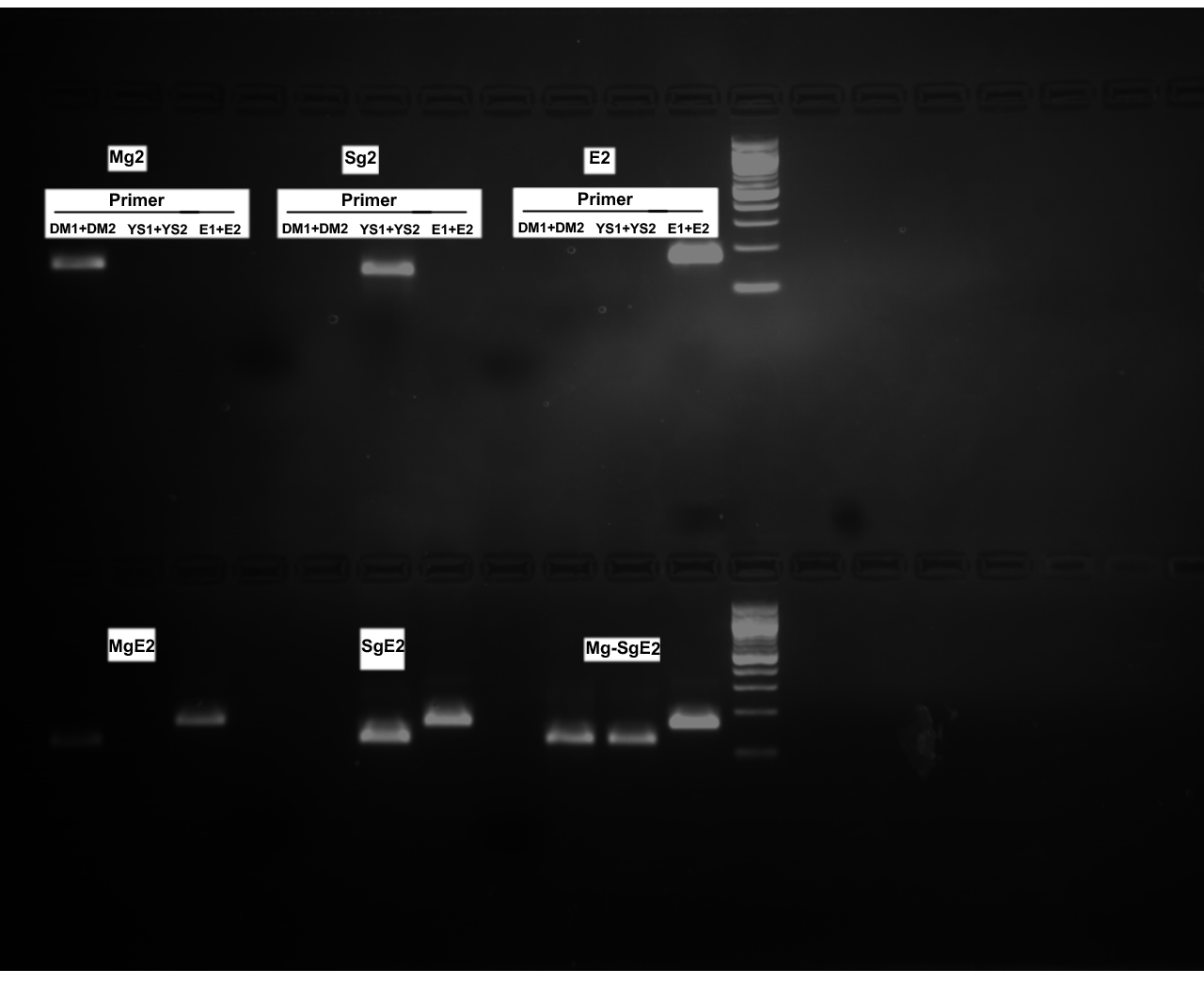

Supplement: Source data 1. [file elife-77584-data1.zip › Source data 28sep/Appendix 1-Figure 1-source data 28sep/Appendix 1-Figure 1-DNA gel of PCR verification 2.tif]

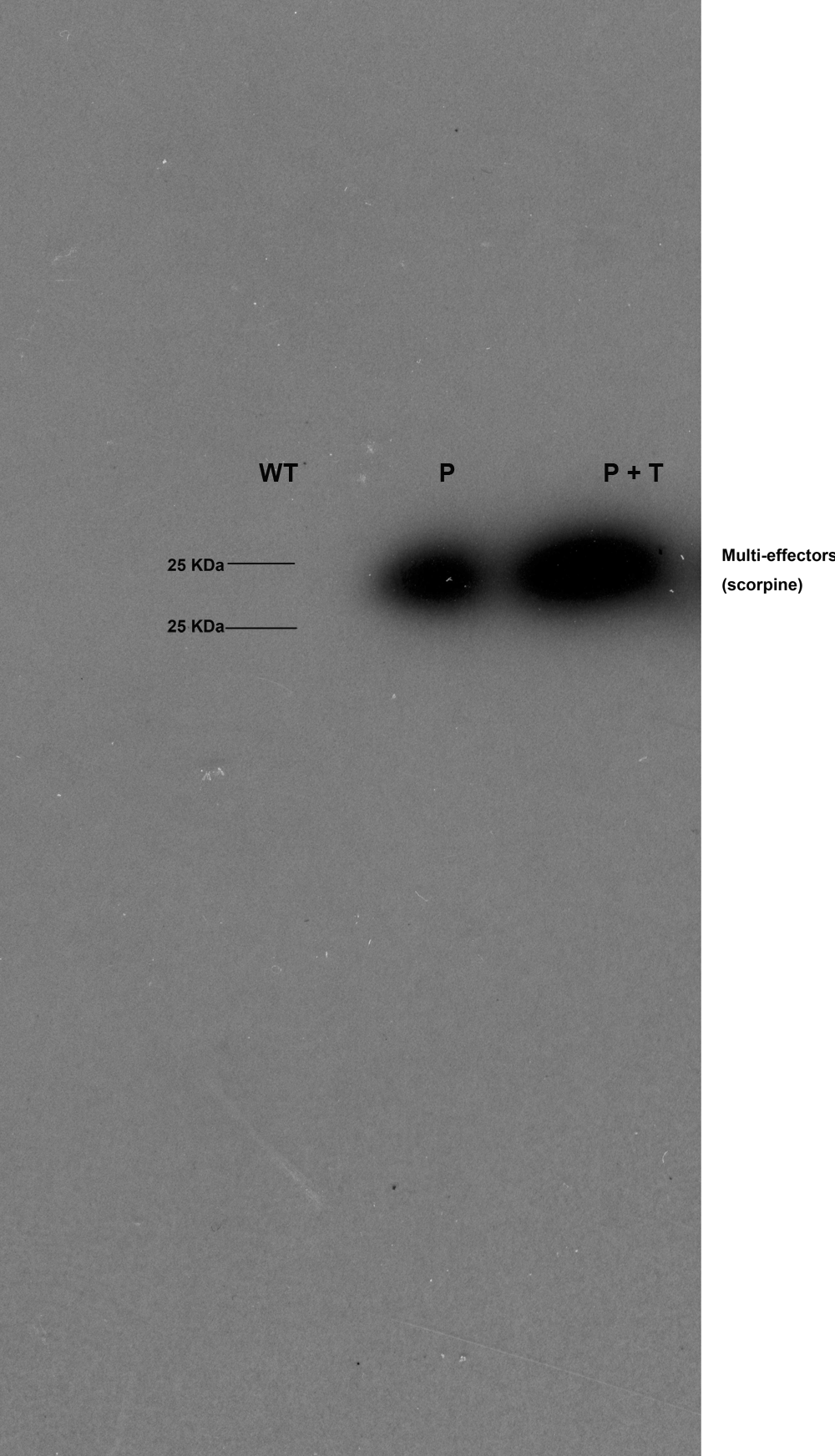

Supplement: Source data 1. [file elife-77584-data1.zip › Source data 28sep/Appendix 1-Figure 6-source data 28sep/Appendix 1-Figure 6-western blot- multi-effectors(Scorpine antibody) in midgut.tif]

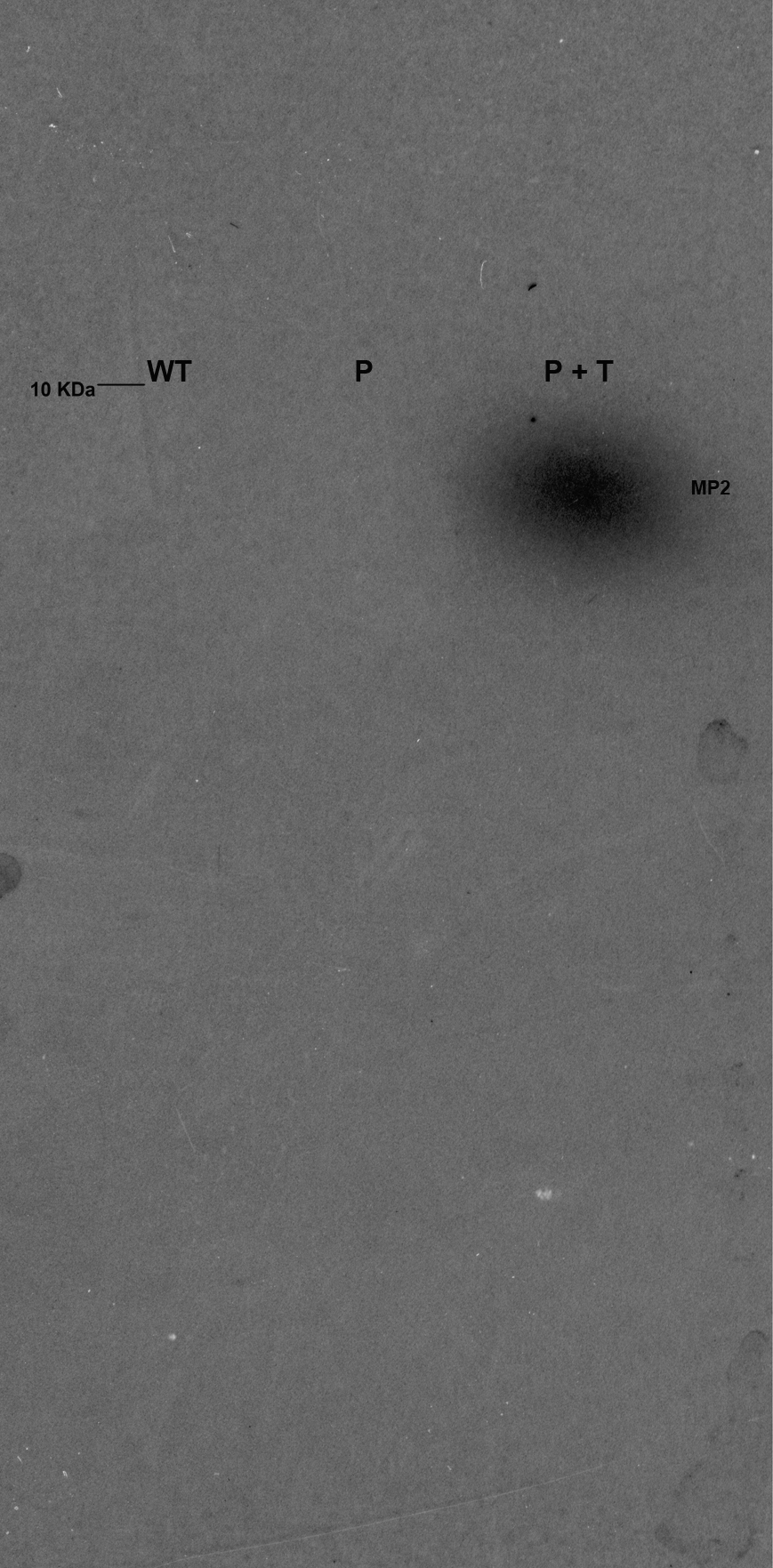

Supplement: Source data 1. [file elife-77584-data1.zip › Source data 28sep/Appendix 1-Figure 6-source data 28sep/Appendix 1-Figure 6-western blot-MP2 in midgut..tif]

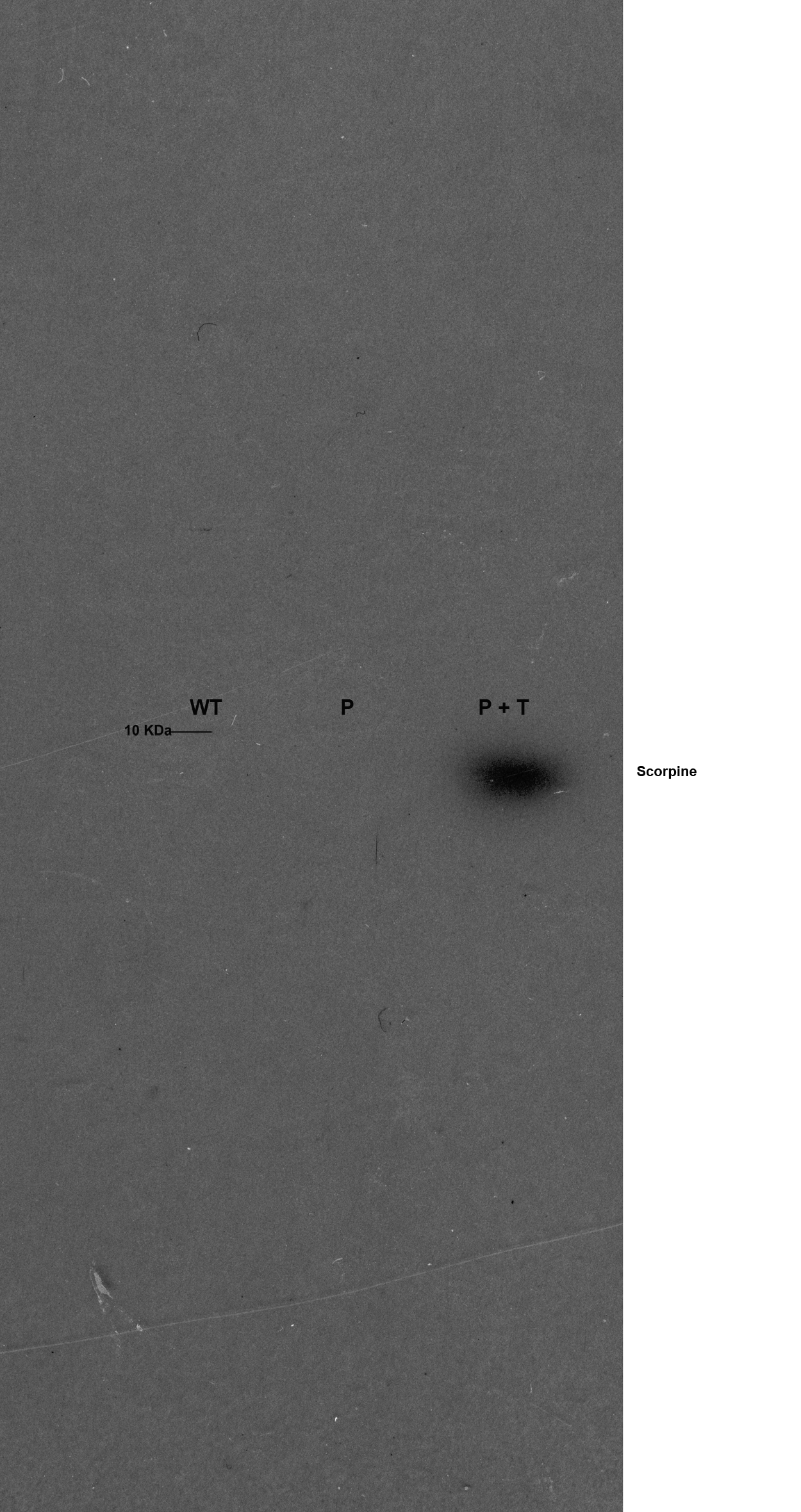

Supplement: Source data 1. [file elife-77584-data1.zip › Source data 28sep/Appendix 1-Figure 6-source data 28sep/Appendix 1-Figure 6-western blot-Scorpine in midgut.tif]

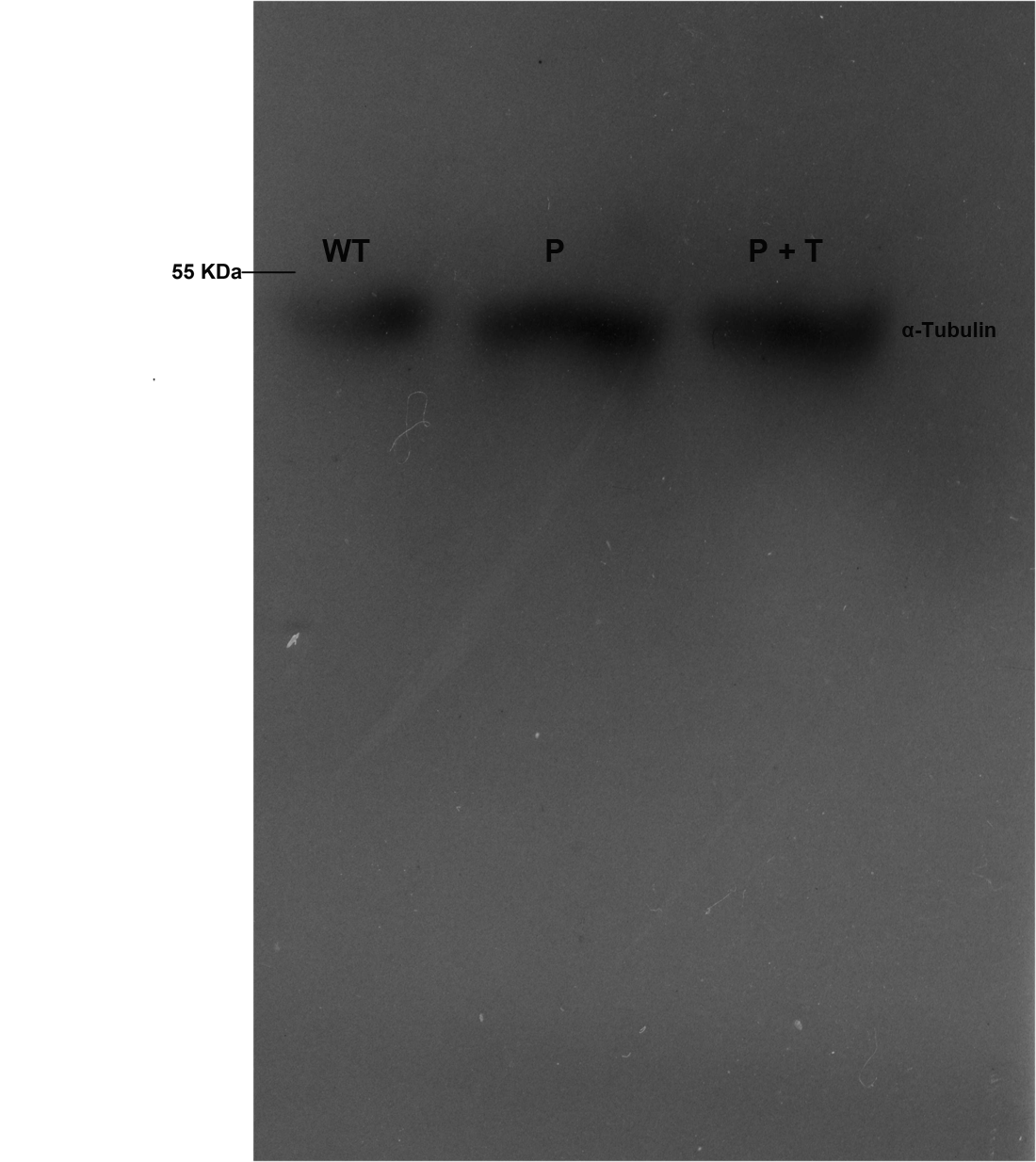

Supplement: Source data 1. [file elife-77584-data1.zip › Source data 28sep/Appendix 1-Figure 6-source data 28sep/Appendix 1-Figure 6-western blot-a┴-Tubulin in midgut..tif]

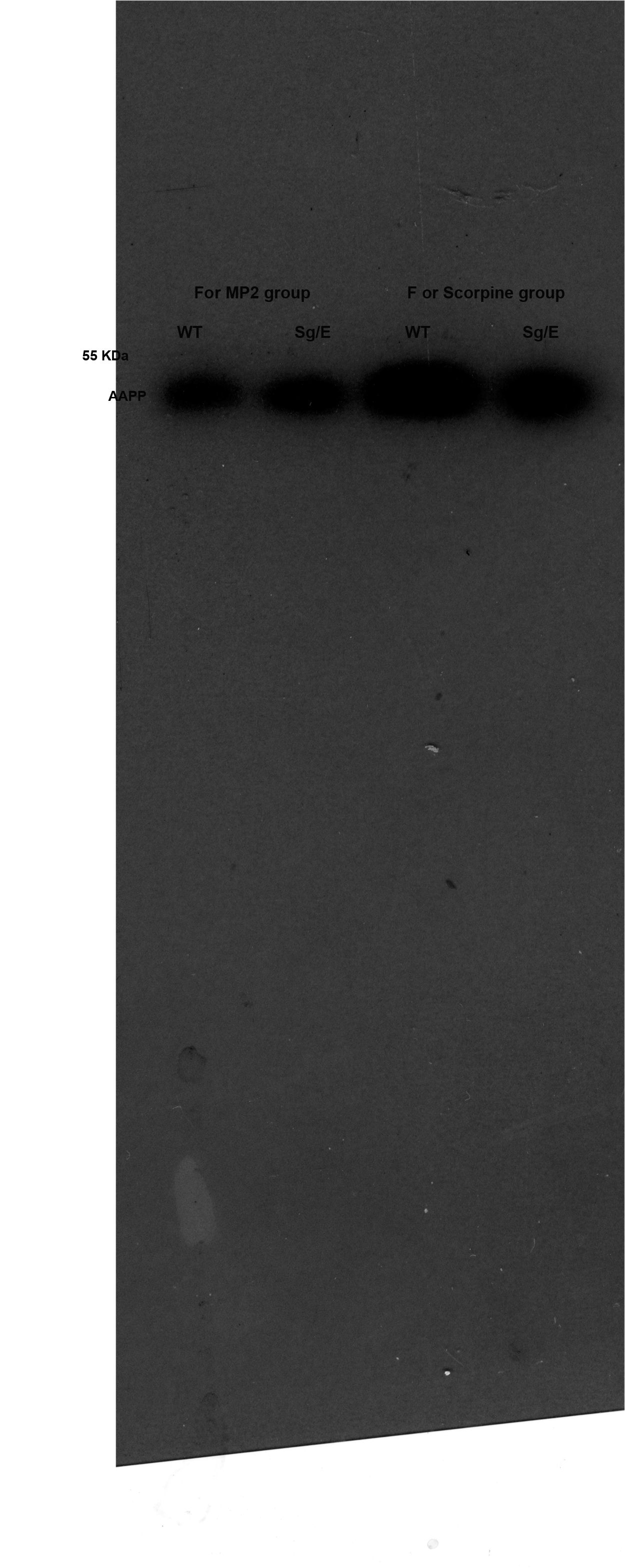

Supplement: Source data 1. [file elife-77584-data1.zip › Source data 28sep/Appendix 1-Figure 7-source data 28sep/Appendix 1-Figure 7-western blot-AAPP in midgut by digestion.tif]

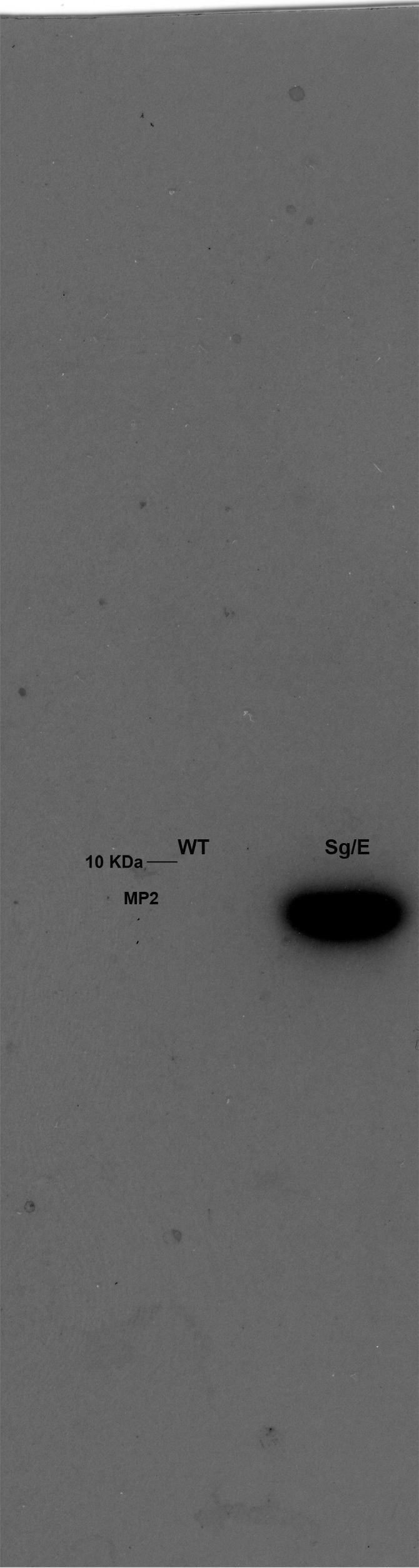

Supplement: Source data 1. [file elife-77584-data1.zip › Source data 28sep/Appendix 1-Figure 7-source data 28sep/Appendix 1-Figure 7-western blot-MP2 in midgut by digestion.tif]

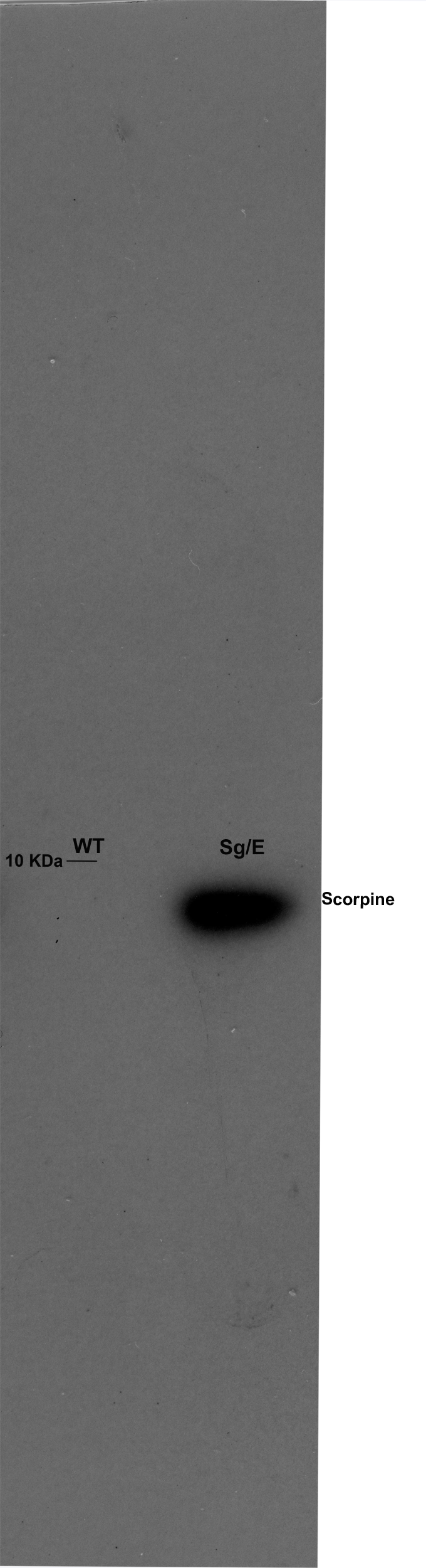

Supplement: Source data 1. [file elife-77584-data1.zip › Source data 28sep/Appendix 1-Figure 7-source data 28sep/Appendix 1-Figure 7-western blot-Scorpine in midgut by digestion.tif]
