# Supplementary material for: Combining transgenesis with paratransgenesis to fight malaria
Source: eLife. 2022 Oct 25;11:e77584. doi: 10.7554/eLife.77584 (PMC9596157; doi:10.7554/eLife.77584)
Supplement: Figure 4—source data 1. — ‘Figure 4BCDE-source data.xlsx’ is the original data of challenge experiment for Figure 4B–E; ‘Figure 4 BCDE-source data-Challenge experiment.pzf’ shows that Figure 4B–E were generated with GraphPad Prism; ‘Figure 4-source data-3 mosquito half infection time calculation by SPSS.spv’ and ‘Figure 4-source data-5 mosquito half infection time calculation by SPSS.spv’ show the calculation of p-value and half-infection time with IBM SPSS version 21 software; ‘Figure 4-source data-3 mosquito half infection time calculation by SPSS.docx’ and ‘Figure 4-source data-3 mosquitoes P value and half infection time.docx’ show the analysis of half-infection time with IBM SPSS, and summary of p-value and half-infection time; ‘Figure 4-source data-5 mosquito half infection time calculation by SPSS.docx’ and ‘Figure 4- source data-5 mosquitoes P value and half infection time.docx’ show the analysis of half-infection time with IBM SPSS, and summary of p-value and half-infection time. [file elife-77584-fig4-data1.zip › Fig 4-source data/Fig4- source data-5 mosquito half infection time calculation by SPSS.docx]

DATASET NAME DataSet1 WINDOW=FRONT.
DATASET ACTIVATE DataSet0.
DATASET CLOSE DataSet1.
KM VAR00002 BY VAR00001
  /STATUS=VAR00003(1)
  /PRINT TABLE MEAN
  /PLOT SURVIVAL.


Kaplan-Meier


Notes	
Output Created	12-FEB-2021 00:24:33	
Comments		
Input	Active Dataset	DataSet0	
	Filter	<none>	
	Weight	<none>	
	Split File	<none>	
	N of Rows in Working Data File	60	
Missing Value Handling	Definition of Missing	User-defined missing values are treated as missing.	
	Cases Used	Statistics are based on all cases with valid data for all variables in the analysis.	
Syntax	KM VAR00002 BY VAR00001
  /STATUS=VAR00003(1)
  /PRINT TABLE MEAN
  /PLOT SURVIVAL.	
Resources	Processor Time	00:00:01.51	
	Elapsed Time	00:00:01.14	


[DataSet0] 


Case Processing Summary	
VAR00001	Total N	N of Events	Censored	
			N	Percent	
.00	15	14	1	6.7%	
1.00	15	11	4	26.7%	
2.00	15	8	7	46.7%	
3.00	15	3	12	80.0%	
Overall	60	36	24	40.0%	


Survival Table	
VAR00001	Time	Status	Cumulative Proportion Surviving at the Time	N of Cumulative Events	N of Remaining Cases	
			Estimate	Std. Error			
.00	1	3.000	1.00	.	.	1	14	
	2	3.000	1.00	.	.	2	13	
	3	3.000	1.00	.800	.103	3	12	
	4	4.000	1.00	.	.	4	11	
	5	4.000	1.00	.667	.122	5	10	
	6	5.000	1.00	.	.	6	9	
	7	5.000	1.00	.	.	7	8	
	8	5.000	1.00	.	.	8	7	
	9	5.000	1.00	.	.	9	6	
	10	5.000	1.00	.333	.122	10	5	
	11	6.000	1.00	.	.	11	4	
	12	6.000	1.00	.200	.103	12	3	
	13	7.000	1.00	.133	.088	13	2	
	14	8.000	1.00	.067	.064	14	1	
	15	14.000	.00	.	.	14	0	
1.00	1	4.000	1.00	.	.	1	14	
	2	4.000	1.00	.867	.088	2	13	
	3	5.000	1.00	.	.	3	12	
	4	5.000	1.00	.733	.114	4	11	
	5	6.000	1.00	.	.	5	10	
	6	6.000	1.00	.	.	6	9	
	7	6.000	1.00	.	.	7	8	
	8	6.000	1.00	.467	.129	8	7	
	9	7.000	1.00	.400	.126	9	6	
	10	9.000	1.00	.333	.122	10	5	
	11	10.000	1.00	.267	.114	11	4	
	12	14.000	.00	.	.	11	3	
	13	14.000	.00	.	.	11	2	
	14	14.000	.00	.	.	11	1	
	15	14.000	.00	.	.	11	0	
2.00	1	4.000	1.00	.933	.064	1	14	
	2	5.000	1.00	.	.	2	13	
	3	5.000	1.00	.800	.103	3	12	
	4	6.000	1.00	.733	.114	4	11	
	5	8.000	1.00	.	.	5	10	
	6	8.000	1.00	.	.	6	9	
	7	8.000	1.00	.533	.129	7	8	
	8	9.000	1.00	.467	.129	8	7	
	9	14.000	.00	.	.	8	6	
	10	14.000	.00	.	.	8	5	
	11	14.000	.00	.	.	8	4	
	12	14.000	.00	.	.	8	3	
	13	14.000	.00	.	.	8	2	
	14	14.000	.00	.	.	8	1	
	15	14.000	.00	.	.	8	0	
3.00	1	3.000	1.00	.	.	1	14	
	2	3.000	1.00	.867	.088	2	13	
	3	6.000	1.00	.800	.103	3	12	
	4	14.000	.00	.	.	3	11	
	5	14.000	.00	.	.	3	10	
	6	14.000	.00	.	.	3	9	
	7	14.000	.00	.	.	3	8	
	8	14.000	.00	.	.	3	7	
	9	14.000	.00	.	.	3	6	
	10	14.000	.00	.	.	3	5	
	11	14.000	.00	.	.	3	4	
	12	14.000	.00	.	.	3	3	
	13	14.000	.00	.	.	3	2	
	14	14.000	.00	.	.	3	1	
	15	14.000	.00	.	.	3	0	


Means and Medians for Survival Time	
VAR00001	Meana	Median	
	Estimate	Std. Error	95% Confidence Interval	Estimate	Std. Error	
			Lower Bound	Upper Bound			
.00	5.533	.686	4.190	6.877	5.000	.365	
1.00	8.267	.977	6.351	10.182	6.000	.773	
2.00	10.067	1.004	8.098	12.035	9.000	.	
3.00	12.000	1.046	9.951	14.049	.	.	
Overall	8.967	.561	7.867	10.067	8.000	1.285	

Means and Medians for Survival Time	
VAR00001	Mediana	
	95% Confidence Interval	
	Lower Bound	Upper Bound	
.00	4.284	5.716	
1.00	4.485	7.515	
2.00	.	.	
3.00	.	.	
Overall	5.482	10.518	

a. Estimation is limited to the largest survival time if it is censored.	
