# Supplementary material for: Combining transgenesis with paratransgenesis to fight malaria
Source: eLife. 2022 Oct 25;11:e77584. doi: 10.7554/eLife.77584 (PMC9596157; doi:10.7554/eLife.77584)
Supplement: Figure 4—source data 1. — ‘Figure 4BCDE-source data.xlsx’ is the original data of challenge experiment for Figure 4B–E; ‘Figure 4 BCDE-source data-Challenge experiment.pzf’ shows that Figure 4B–E were generated with GraphPad Prism; ‘Figure 4-source data-3 mosquito half infection time calculation by SPSS.spv’ and ‘Figure 4-source data-5 mosquito half infection time calculation by SPSS.spv’ show the calculation of p-value and half-infection time with IBM SPSS version 21 software; ‘Figure 4-source data-3 mosquito half infection time calculation by SPSS.docx’ and ‘Figure 4-source data-3 mosquitoes P value and half infection time.docx’ show the analysis of half-infection time with IBM SPSS, and summary of p-value and half-infection time; ‘Figure 4-source data-5 mosquito half infection time calculation by SPSS.docx’ and ‘Figure 4- source data-5 mosquitoes P value and half infection time.docx’ show the analysis of half-infection time with IBM SPSS, and summary of p-value and half-infection time. [file elife-77584-fig4-data1.zip › Fig 4-source data/Fig4- source data-3 mosquito half infection time calculation by SPSS.docx]

KM VAR00002 BY VAR00001
  /STATUS=VAR00003(1)
  /PRINT TABLE MEAN
  /PLOT SURVIVAL
  /TEST LOGRANK BRESLOW
  /COMPARE OVERALL POOLED.


Kaplan-Meier


Notes	
Output Created	08-JAN-2021 15:00:47	
Comments		
Input	Active Dataset	DataSet0	
	Filter	<none>	
	Weight	<none>	
	Split File	<none>	
	N of Rows in Working Data File	32	
Missing Value Handling	Definition of Missing	User-defined missing values are treated as missing.	
	Cases Used	Statistics are based on all cases with valid data for all variables in the analysis.	
Syntax	KM VAR00002 BY VAR00001
  /STATUS=VAR00003(1)
  /PRINT TABLE MEAN
  /PLOT SURVIVAL
  /TEST LOGRANK BRESLOW
  /COMPARE OVERALL POOLED.	
Resources	Processor Time	00:00:00.19	
	Elapsed Time	00:00:00.17	


[DataSet0] 


Case Processing Summary	
VAR00001	Total N	N of Events	Censored	
			N	Percent	
.00	15	15	0	0.0%	
1.00	11	11	0	0.0%	
2.00	5	5	0	0.0%	
3.00	1	1	0	0.0%	
Overall	32	32	0	0.0%	


Survival Table	
VAR00001	Time	Status	Cumulative Proportion Surviving at the Time	N of Cumulative Events	N of Remaining Cases	
			Estimate	Std. Error			
.00	1	4.000	1.00	.	.	1	14	
	2	4.000	1.00	.	.	2	13	
	3	4.000	1.00	.	.	3	12	
	4	4.000	1.00	.733	.114	4	11	
	5	5.000	1.00	.	.	5	10	
	6	5.000	1.00	.	.	6	9	
	7	5.000	1.00	.	.	7	8	
	8	5.000	1.00	.	.	8	7	
	9	5.000	1.00	.	.	9	6	
	10	5.000	1.00	.333	.122	10	5	
	11	6.000	1.00	.	.	11	4	
	12	6.000	1.00	.	.	12	3	
	13	6.000	1.00	.133	.088	13	2	
	14	8.000	1.00	.067	.064	14	1	
	15	11.000	1.00	.000	.000	15	0	
1.00	1	5.000	1.00	.	.	1	10	
	2	5.000	1.00	.	.	2	9	
	3	5.000	1.00	.	.	3	8	
	4	5.000	1.00	.636	.145	4	7	
	5	6.000	1.00	.	.	5	6	
	6	6.000	1.00	.455	.150	6	5	
	7	8.000	1.00	.	.	7	4	
	8	8.000	1.00	.	.	8	3	
	9	8.000	1.00	.182	.116	9	2	
	10	10.000	1.00	.091	.087	10	1	
	11	12.000	1.00	.000	.000	11	0	
2.00	1	5.000	1.00	.	.	1	4	
	2	5.000	1.00	.	.	2	3	
	3	5.000	1.00	.400	.219	3	2	
	4	7.000	1.00	.200	.179	4	1	
	5	10.000	1.00	.000	.000	5	0	
3.00	1	6.000	1.00	.000	.000	1	0	


Means and Medians for Survival Time	
VAR00001	Meana	Median	
	Estimate	Std. Error	95% Confidence Interval	Estimate	Std. Error	
			Lower Bound	Upper Bound			
.00	5.533	.477	4.599	6.468	5.000	.304	
1.00	7.091	.707	5.706	8.476	6.000	.991	
2.00	6.400	.980	4.480	8.320	5.000	.	
3.00	6.000	.000	6.000	6.000	6.000	.	
Overall	6.219	.372	5.489	6.948	5.000	.297	

Means and Medians for Survival Time	
VAR00001	Mediana	
	95% Confidence Interval	
	Lower Bound	Upper Bound	
.00	4.404	5.596	
1.00	4.058	7.942	
2.00	.	.	
3.00	.	.	
Overall	4.418	5.582	

a. Estimation is limited to the largest survival time if it is censored.	


Overall Comparisons	
	Chi-Square	df	Sig.	
Log Rank (Mantel-Cox)	3.684	3	.298	
Breslow (Generalized Wilcoxon)	4.777	3	.189	

Test of equality of survival distributions for the different levels of VAR00001.	
