# Supplementary material for: Combining transgenesis with paratransgenesis to fight malaria
Source: eLife. 2022 Oct 25;11:e77584. doi: 10.7554/eLife.77584 (PMC9596157; doi:10.7554/eLife.77584)
Supplement: Figure 4—source data 1. — ‘Figure 4BCDE-source data.xlsx’ is the original data of challenge experiment for Figure 4B–E; ‘Figure 4 BCDE-source data-Challenge experiment.pzf’ shows that Figure 4B–E were generated with GraphPad Prism; ‘Figure 4-source data-3 mosquito half infection time calculation by SPSS.spv’ and ‘Figure 4-source data-5 mosquito half infection time calculation by SPSS.spv’ show the calculation of p-value and half-infection time with IBM SPSS version 21 software; ‘Figure 4-source data-3 mosquito half infection time calculation by SPSS.docx’ and ‘Figure 4-source data-3 mosquitoes P value and half infection time.docx’ show the analysis of half-infection time with IBM SPSS, and summary of p-value and half-infection time; ‘Figure 4-source data-5 mosquito half infection time calculation by SPSS.docx’ and ‘Figure 4- source data-5 mosquitoes P value and half infection time.docx’ show the analysis of half-infection time with IBM SPSS, and summary of p-value and half-infection time. [file elife-77584-fig4-data1.zip › Fig 4-source data/Fig4- source data-3 mosquitoes P value and half infection time.docx]

WT&paratransgenesis P=0.0066

WT&transgenesis P< 0.0001

WT&paratransgenesis+transgenesis P< 0.0001

WT=5.533+0.477

paratransgenesis=7.091+0.707
